# Estimation of genetic parameters of pig reproductive traits

**DOI:** 10.3389/fvets.2023.1172287

**Published:** 2023-06-21

**Authors:** Yiting Yang, Mailin Gan, Xidi Yang, Peng Zhu, Yi Luo, Bin Liu, Kangping Zhu, Wenqiang Cheng, Lei Chen, Ye Zhao, Lili Niu, Yan Wang, Hui Zhang, Jingyong Wang, Linyuan Shen, Li Zhu

**Affiliations:** ^1^Farm Animal Genetic Resources Exploration and Innovation Key Laboratory of Sichuan Province, Sichuan Agricultural University, Chengdu, China; ^2^Key Laboratory of Livestock and Poultry Multi-Omics, Ministry of Agriculture and Rural Affairs, College of Animal and Technology, Sichuan Agricultural University, Chengdu, China; ^3^Sichuan Dekon Livestock Foodstuff Group, Chengdu, China; ^4^National Animal Husbandry Service, Beijing, China; ^5^Sichuan Center for Animal Disease Control, Chengdu, China; ^6^Chongqing Academy of Animal Science, Chongqing, China

**Keywords:** reproductive traits, genetic parameters, animal mixed linear model, influencing factors, pig breeding

## Abstract

**Introduction:**

In this study, we aimed to estimate the genetic parameters of the reproductive traits in three popular commercial pig breeds: Duroc, Landrace, and Yorkshire. Additionally, we evaluated the factors that influence these traits.

**Method:**

We collected data from a large number of litters, including 1,887 Duroc, 21,787 Landrace, and 74,796 Yorkshire litters. Using the ASReml-R software to analyze 11 traits, which included: total number of pigs born (TNB); number of piglets born alive (NBA); number of piglets born healthy (NBH); number of piglets born weak (NBW); number of new stillborn piglets (NS); number of old stillborn piglets (OS); number of piglets born with malformation (NBM); number of mummified piglets (NM); total litter birthweight (LBW); litter average weight (LAW); duration of gestational period (GP). We investigated the effects of 4 fixed factors on the genetic parameters of these traits.

**Results:**

Among the 11 reproductive-related traits, the gestational period belonged to the medium heritability traits (0.251–0.430), while remaining traits showed low heritability, ranging from 0.005 to 0.159. TNB, NBA, NBH, LBW had positive genetic correlation (0.737 ~ 0.981) and phenotype correlation (0.711 ~ 0.951). There was a negative genetic correlation between NBW and LAW (−0.452 ~ −0.978) and phenotypic correlation (−0.380 ~ −0.873). LBW was considered one of the most reasonable reproductive traits that could be used for breeding improvement. Repeatability of the three varieties was within the range of 0.000–0.097. In addition, the fixed effect selected in this study had a significant effect on Landrace and Yorkshire (*p* < 0.05).

**Discussion:**

We found a positive correlation between LBW and TNB, NBA, and NBH, suggesting the potential for multi-trait association breeding. Factors such as farm, farrowing year, breeding season, and parity should be taken into consideration in practical production, as they may impact the reproductive performance of breeding pigs.

## 1. Introduction

Reproductive traits are one of the most economically important traits in pig farming, which has significance for evaluating the reproductive efficiency of purebred sows and pig breeding selection (1). Improved reproductive efficiency is associated with more economic benefits for pig farmers (2). In breeding and selection of purebred sows, accurately determining estimated breeding value (EBV) of reproductive traits can optimize the breeding program and predict the genetic response (3). Therefore, estimating the genetic parameters associated with reproductive traits is an indispensable component of assessing population characteristics and improving breeding programs.

Over the years, research has focused on determining EBVs of reproductive traits, and genetic parameters for farrowing traits have been estimated (4, 5) over the last century for different commercial populations with limited real-world data. Oh et al. (6) showed that low genetic correlation between the first parity and the subsequent parity for different reproductive traits. Ogewa et al. (7) used a single-trait animal model to estimate the genetic parameters of six reproductive traits in Landrace and Yorkshire. However, due to differences in data volume, algorithms, target reproductive traits, and population classification, different results and degrees of accuracy were obtained in previous studies. Therefore, the use of multiple models to accurately fit the variance of components and estimate the genetic parameters for comprehensively measuring reproductive traits is of great significance for guiding the genetic selection of pigs.

In order to enhance breeding decisions and improve population reproductive efficiency, the aim of this study was to determine 11 reproductive-related phenotypic traits: total number of pigs born (TNB); number of piglets born alive (NBA); number of piglets born healthy (NBH); number of piglets born weak (NBW); number of new stillborn piglets (NS); number of old stillborn piglets (OS); number of piglets born with malformation (NBM); number of mummified piglets (NM); total litter birthweight (LBW); litter average weight (LAW); duration of gestational period (GP), to build a variety of models for estimating genetic parameters. In addition, the different model and fixed effects were tested by statistical methods to verify the accuracy of estimated heritability in order to explore the influencing factors.

## 2. Materials and methods

### 2.1. Description of phenotypic data

The data utilized in this study were obtained from 11 breeding pig farms of the same company located in Southwest China, where Canadian breeding lines were raised. It is important to note that there is genetic exchange among the breeding pig populations across different farms. A total of 98,470 litters of sow farrowing records obtained within the years of 2016–2022, among which were 1,887 Duroc, 21,787 Landrace, and 74,796 Yorkshire. In addition, we collected a total of 48,434 pedigree data from 3 breeds, each of which consisted of 8 generations. Reproductive trait records were TNB, NBA, NBH, NBW, NS, OS, NBM, NM, LBW, LAW, and GP; NBA was the number of piglets born alive per litter within 24 h of birth; NS referred to piglets with no vital signs after parturition; OS referred to stillborn piglets obtained from sows was yellow, old and dark in color, but the fetus is not mummified, that is, the piglet died in the mother for a period of time; LAW was the average of total litter weight within 24 h after birth, excluding stillborn and mummified piglets.

### 2.2. Data processing and software analysis

Depending on data distribution and prior knowledge, TNB or NBA data with values <3 or >30 were discarded. LAW records <0.3 kg or >3 kg were excluded from this study. Gp values were limited to 104 ~ 124 days. Duplicating pedigree records were removed, individuals with phenotype data were completed, no pedigree records through self-written python scripts were recorded, and completes the number of zero was assigned to parental individual information.

The variance of components of 11 reproductive traits using the ‘ASReml-R’ (8) R package, and the vpredict function was used for determining heritability calculations. This package was designed to fit linear mixed models, especially for plant and animal breeding, to estimate the genetic parameters using restricted maximum likelihood.

### 2.3. Statistical models

#### 2.3.1. Simple animal models

In the present study, single-trait and two-trait animal models were used to estimate the variance of components of 11 reproductive traits. Model1 formulas were determined as follows:

y=μ+Xf+Za+e

where *y* is the vector of phenotypic records; *f* is the fixed effect vector of the year of gestation, parturition season, farm, and parity, including gestation year effect, gestation season effect, parity effect and farm effect; *ɑ* is the additive genetic effect ~(0, $A\sigma_{a}^{2}$ ); *e* is the residual effect vector ~(0, $I\sigma_{e}^{2}$ ); and *X* and *Z* are the corresponding matrices. We utilized assumed (co)variance matrices to account for random and residual effects in our analysis. Specifically, we employed Var(*a*) = G0 ⊗ *A* to represent the additive genetic (co)variance matrix between traits, where G0 captures the additive genetic relationships among individuals as indicated by Wright’s numerator relationship matrix (A). Similarly, we used Var(*e*) = R0 ⊗ *I* to represent the residual (co)variance matrix between traits, where R0 represents the residual covariance matrix and *I* represents the identity matrix.

Breeding seasons of the sows on each breed were defined into spring (March–May), summer (June–August), autumn (September–November), and winter (December–February). Breeding records of all three varieties were purebred. The formula for calculating heritability was:

$$h^{2}=\frac{\sigma_{a}^{2}}{\sigma_{a}^{2}+\sigma_{e}^{2}}$$

where *h*^2^ is heritability, *σa*^2^ is additive genetic variance, and *σe*^2^ is residual variance.

Genetic and phenotypic correlations were calculated using the following formula:

$$r_{G}=\frac{{\mathrm{COV}}_{G_{XY}}}{\sqrt{\sigma_{G_{X}}^{2}\sigma_{G_{Y}}^{2}}}r_{P}=\frac{{\mathrm{COV}}_{P_{XY}}}{\sqrt{\sigma_{P_{X}}^{2}\sigma_{P_{Y}}^{2}}}$$

where $r_{G}$ is the genetic correlation between traits *X* and *Y*; ${\mathrm{COV}}_{G_{XY}}$ is the genetic covariance matrix of traits *X* and *Y*; $\sigma_{G_{X}}$ and $\sigma_{G_{Y}}$ are the genetic standard deviation of traits *X* and *Y*; $r_{P}$ is the phenotypic correlation between traits *X* and *Y*; ${\mathrm{COV}}_{P_{XY}}$ is the phenotypic covariance matrix of traits *X* and *Y*; $\sigma_{P_{X}}$ and $\sigma_{P_{Y}}$ are the phenotype standard deviation of traits *X* and *Y*.

#### 2.3.2. Repeatability model

Previously, we calculated the proportion of additive variance in the simple animal model. In contrast, for the repeatability model, we calculated the proportions of additive variance and permanent environmental effects variance separately, taking into account the influence of permanent environmental effects. The repeatability model is formulated as follows:

y=Xf+Za+Wp+e

where *f* is the fixed effect; *α* is the additive genetic effect; p is the permanent environmental effect; e is the residual effect; and *X*, *Z*, and *W* are the corresponding structural matrices.

The formulas for calculating heritability and repeatability were:

$$h^{2}=\frac{\sigma_{a}^{2}}{\sigma_{p}^{2}}\mathrm{rep}=\frac{\sigma_{pe}^{2}}{\sigma_{p}^{2}}$$

where *h*^2^ is the heritability; rep is the repeatability; $\;\sigma_{a}^{2}$ is the additive genetic variance; $\sigma_{pe}^{2}$ is the permanent environmental variance; $\sigma_{p}^{2}$ is the total phenotypic variance, which is the sum of $\sigma_{a}^{2}\;\sigma_{pe}^{2}\;\sigma_{e}^{2}$ , and $\sigma_{e}^{2}$ is the residual variance.

### 2.4. Model validation and fixed effects testing

The model was tested using the REML likelihood ratio test (LRT) in ASReml-R, and fixed effects testing was conducted using the Wald function (Supplementary material). In the simple animal model1, changes in genetic correlation were determined using the us function of additive diag function (co-correlation was not considered) to test the significance using LRT, and changes in episodic correlation were determined using the us function of additive and residual diag function to determine significance. In the repeatability model, model2 was very similar to the model1. The comparison between these two models is aimed at calculating heritability more accurately (i.e., by choosing between including or excluding permanent environmental effects in the model).

## 3. Results

### 3.1. Statistics of quantitative traits of phenotypic values

The table (Table 1) presents the mean, standard deviation, maximum and minimum values of the 11 reproductive traits in Duroc, Landrace, and Yorkshire. TNB, NBA, NBH, and LAW in Duroc sows were lower than in the other two pig breeds; OS and NM were higher than in the other two breeds, which showed that Duroc pigs have relatively weak reproductive ability. For Landrace and Yorkshire pigs, as the intermediate male and terminal female parent in the commercial pig crossbreeding (9), their reproductive performance was selected as the main selection object in early breeding. TNB in Yorkshire sows was 13.98 ± 3.31, which was significantly different from TNB in Landrace sows (12.7 ± 3.36). Moreover, TNB, NBA, NBH, NBW, NS, and LBW in Yorkshire sows were consistently higher than in the other two pig breeds.

**Table 1:** Descriptive statistics of reproductive traits in farrowing sows.

Trait	Duroc				Landrace				Yorkshire			
	Mean	SD	Min	Max	Mean	SD	Min	Max	Mean	SD	Min	Max
TNB	10.18	2.85	1	25	12.70	3.36	1	25	13.98	3.31	1	30
NBA	8.81	2.63	1	23	11.73	3.19	1	22	12.81	3.11	1	26
NBH	8.29	2.45	1	23	11.13	3.00	0	21	11.92	2.84	0	23
NBW	0.42	0.79	0	7	0.48	0.88	0	11	0.84	1.25	0	17
NS	0.46	0.77	0	5	0.46	0.85	0	12	0.55	0.94	0	18
OS	0.30	0.67	0	6	0.25	0.72	0	17	0.29	0.74	0	16
NBM	0.11	0.37	0	4	0.12	0.49	0	11	0.05	0.28	0	11
NM	0.61	1.07	0	10	0.27	0.68	0	12	0.33	0.77	0	18
LBW	12.40	3.56	0.7	23.8	16.92	4.48	0.6	38.4	17.29	4.16	0.4	41.5
LAW	1.43	0.24	0.4	2.75	1.47	0.24	0.4	3	1.37	0.20	0.3	3
GP	115.61	1.20	108	119	115.85	1.45	106	124	114.10	1.48	104	124

Mean, arithmetical mean; SD, standard deviation; Min, minimum value; Max, maximum value.

### 3.2. Estimation of heritability of reproductive traits in single-trait animal models

Heritability estimated based on single-trait animal models are shown in Table 2. Heritability was 0.033–0159 for Duroc, 0.031–0.159 for Landrace, and 0.009–0.196 for Yorkshire. Interestingly, the gestation period showed medium heritability, i.e., 0.43 ± 0.031, 0.282 ± 0.01, 0.388 ± 0.005 for Duroc, Landrace, and Yorkshire, respectively. These findings were similar to those reported for dairy cows (10), which revealed that due to the absence of permanent environmental effects in the single-trait animal model, heritability is relatively high (11). The low heritability values suggest that direct selection for most of the evaluated reproductive traits would lead to only marginal annual genetic progress. In addition, heritability of NS, OS, NBM, and NM was within the range of 0.009–0.084, thus showing a very low proportion of additive inheritance, which has significance for pig breeding. The standard error of heritability of TNB, NBA, and NBH was 0.005–0.009 between Landrace and Yorkshire pig breeds. The lower standard errors observed in these two breeds compared to Duroc suggest that the large amount of data allows additive genetic variance to effectively represent the population level with minimal sampling error.

**Table 2:** Estimates of heritability for reproductive traits in three pig breeds based on the single-trait animal model.

Trait	Item	Duroc			Landrace			Yorkshire		
		Component	SE	*h* ^2^	Component	SE	*h* ^2^	Component	SE	*h* ^2^
TNB	$\sigma_{a}^{2}$	1.280	0.241	0.159 ± 0.027	1.722	0.111	0.153 ± 0.009	2.111	0.064	0.196 ± 0.005
	$\sigma_{e}^{2}$	6.734	0.250		9.544	0.105		8.678	0.052	
NBA	$\sigma_{a}^{2}$	1.009	0.204	0.145 ± 0.027	1.628	0.103	0.159 ± 0.009	1.869	0.057	0.193 ± 0.005
	$\sigma_{e}^{2}$	5.939	0.221		8.634	0.095		7.812	0.047	
NBH	$\sigma_{a}^{2}$	0.820	0.169	0.137 ± 0.026	1.205	0.085	0.133 ± 0.009	1.405	0.046	0.173 ± 0.005
	$\sigma_{e}^{2}$	5.148	0.191		7.834	0.086		6.710	0.040	
NBW	$\sigma_{a}^{2}$	0.051	0.014	0.082 ± 0.022	0.069	0.006	0.093 ± 0.007	0.200	0.007	0.134 ± 0.004
	$\sigma_{e}^{2}$	0.570	0.021		0.676	0.007		1.291	0.008	
NS	$\sigma_{a}^{2}$	0.041	0.013	0.071 ± 0.021	0.025	0.004	0.035 ± 0.005	0.061	0.003	0.073 ± 0.004
	$\sigma_{e}^{2}$	0.529	0.019		0.682	0.007		0.775	0.004	
OS	$\sigma_{a}^{2}$	0.037	0.011	0.084 ± 0.023	0.010	0.002	0.019 ± 0.004	0.013	0.001	0.024 ± 0.002
	$\sigma_{e}^{2}$	0.405	0.015		0.498	0.005		0.524	0.003	
NBM	$\sigma_{a}^{2}$	0.005	0.002	0.033 ± 0.016	0.008	0.001	0.033 ± 0.005	0.001	0.000	0.009 ± 0.002
	$\sigma_{e}^{2}$	0.136	0.005		0.225	0.002		0.078	0.000	
NM	$\sigma_{a}^{2}$	0.085	0.023	0.077 ± 0.02	0.014	0.002	0.031 ± 0.005	0.018	0.002	0.031 ± 0.003
	$\sigma_{e}^{2}$	1.022	0.037		0.439	0.005		0.572	0.003	
LBW	$\sigma_{a}^{2}$	1.557	0.331	0.128 ± 0.025	2.842	0.185	0.146 ± 0.009	3.018	0.095	0.181 ± 0.005
	$\sigma_{e}^{2}$	10.624	0.391		16.596	0.182		13.631	0.082	
LAW	$\sigma_{a}^{2}$	0.006	0.001	0.117 ± 0.024	0.008	0.000	0.157 ± 0.009	0.007	0.000	0.186 ± 0.005
	$\sigma_{e}^{2}$	0.046	0.002		0.043	0.000		0.031	0.000	
GP	$\sigma_{a}^{2}$	0.637	0.069	0.43 ± 0.031	0.594	0.026	0.282 ± 0.01	0.865	0.017	0.388 ± 0.005
	$\sigma_{e}^{2}$	0.846	0.033		1.511	0.017		1.363	0.008	

SE, standard error; *h*^2^, heritability.

### 3.3. Genetic correlation and phenotypic correlation

**Figure 1:**
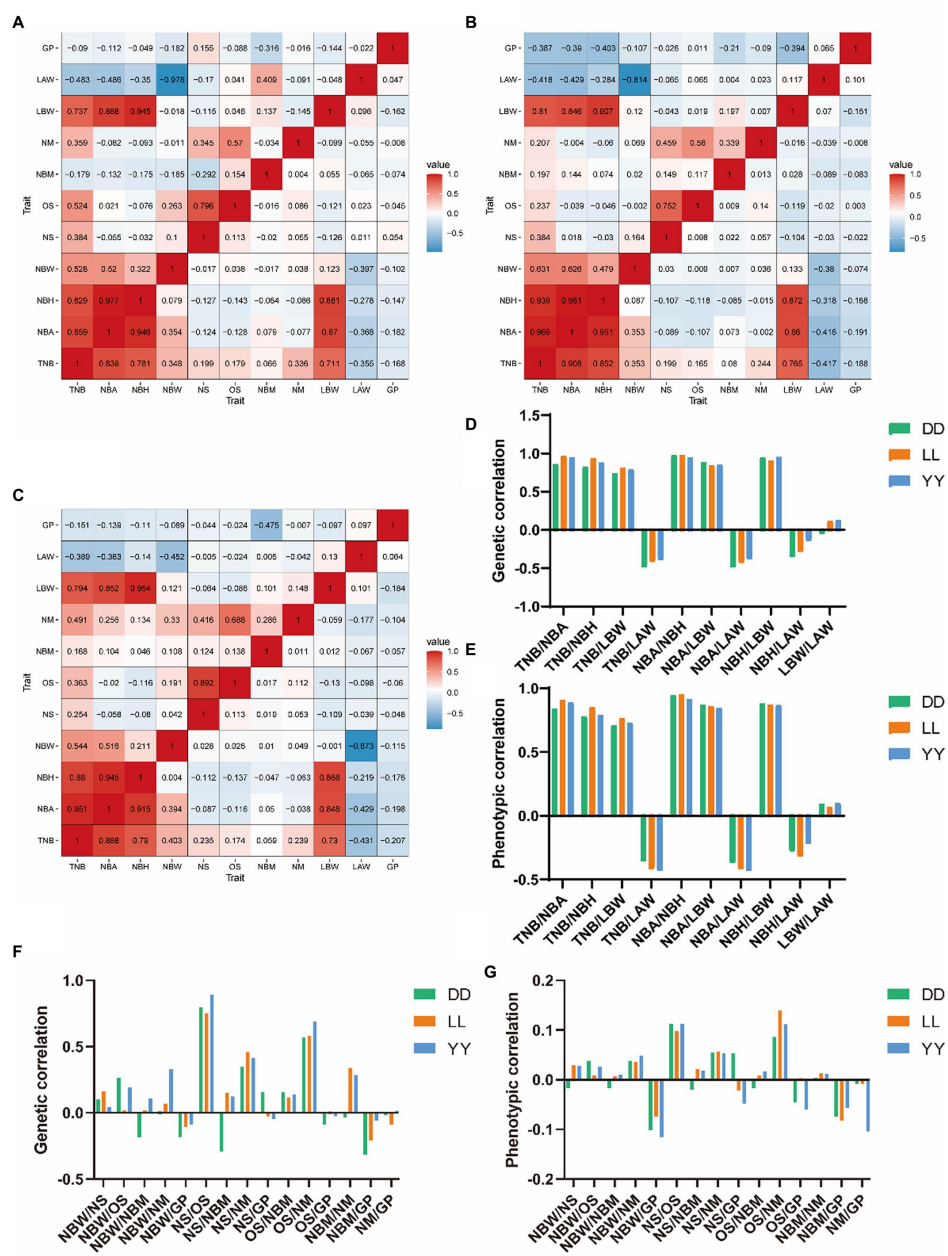
Genetic and phenotypic correlations among evaluated traits: total number of pigs born (TNB); number of piglets born alive (NBA); number of piglets born healthy (NBH); number of piglets born weak (NBW); number of new stillborn piglets (NS); number of old stillborn piglets (OS); number of piglets born with malformation (NBM); number of mummified piglets (NM); total litter birthweight (LBW); litter average weight (LAW); duration of gestational period (GP). Heatmap of genetic correlation and phenotypic correlation in Duroc **(A)**, Landrace **(B)**, and Yorkshire **(C)** (the upper part of the triangle refers to genetic correlation coefficients; the lower part of the triangle refers to phenotypic correlation coefficients), **(D)** genetic correlation of the three pig breeds expected to improve the evaluated traits, **(E)** phenotypic correlation for three pig breeds expected to improve traits, **(F)** genetic correlation coefficients of three pig breeds expected to reduce traits, and **(G)** phenotypic correlations for the three pig breeds expected to reduce traits.

Large sample sizes are often required to accurately estimate genetic correlations in animal breeding, since they are often subjected to large sampling errors (12). In our study, genetic correlations and phenotypic correlations of three pig breeds are shown in Figures 1A–C. LRT of the multi-trait animal model between Landrace and Duroc breeds revealed that most genetic correlations and phenotypic correlations existed. Both genetic correlation and phenotypic correlation, LBW is positively correlated with TNB, NBA, and NBH, within the range of 0.737–0.954, which provided a new idea for multi-trait association breeding. In contrast, LAW was negatively correlated with TNB, NBA, and NBH in both genetic and phenotypic correlations. Moreover, the greater the litter size, the lower the mean litter weight, which was also in line with common sense. In a multi-trait animal model, it was found that although heritability was moderate, it exhibited negative correlations with most reproductive traits in both genetic and phenotypic correlations.

Furthermore, the 11 reproductive traits evaluated in the present study were classified into two groups: (i) genetic correlation and phenotypic correlation of traits that are expected to be improved during production, such as TNB, NBA, NBH, LAW, and LBW and (ii) genetic correlation and phenotypic correlation of traits that are not expected to be improved during production, NBW, NS, OS, NBM, and GP. Considering the first group, a very high consistency in genetic correlation and phenotypic correlation was found in the three pig breeds (Figures 1D,E), which was also in line with expected results. However, the second group showed that Landrace and Yorkshire pigs had a very high consistency in genetic correlation and phenotypic correlation, which may be caused by the lower selection pressure of Droc based on reproductive traits compared with the other two pig breeds.

### 3.4. Estimation of repeatability

The reproductive traits selected in this study are considered repeatable measurable traits. When estimating heritability, the value of permanent environmental effects was calculated by the constructed repeatability model, thus yielding a more accurate estimate of the additive variance ratio. Repeatability results estimated with the optimal model are shown in Table 3; TNB, NBA, and NBH were found within the range of 0.031–0.097, whereas NBW, NS, OS, and NBM were within the range of 0.009–0.087. The repeatability of NM in Duroc pigs was close to 0.000, which indicates that the permanent environmental effect accounted for a very low proportion of NM for Duroc pigs, but might also be related to the variability in the Duroc dataset. LAW, LBW, and GP’s repeatability were between 0.017 ~ 0.087.

**Table 3:** Optimal model selection and estimation of repeatability for three pig breeds (Duroc, Landrace, and Yorkshire).

Trait	Duroc		Landrace		Yorkshire	
	Rep	SE	Rep	SE	Rep	SE
TNB	0.031	0.033	0.079	0.010	0.094	0.006
NBA	0.079	0.034	0.081	0.010	0.097	0.006
NBH	0.032	0.033	0.078	0.010	0.094	0.005
NBW	0.029	0.028	0.037	0.008	0.048	0.005
NS	0.080	0.022	0.025	0.007	0.027	0.004
OS	0.087	0.022	0.009	0.007	0.016	0.004
NBM	0.041	0.020	0.036	0.008	0.017	0.003
NM	0.000	0.000	0.014	0.007	0.019	0.004
LBW	0.035	0.032	0.073	0.010	0.087	0.005
LAW	0.017	0.029	0.062	0.010	0.070	0.005
GP	0.044	0.041	0.043	0.012	0.069	0.007

Rep, repeatability; SE, standard error.

Heritability results estimated with the repeatability model are shown in Table 4 (Supplementary Tables S4–S6). Respectively, heritability of TNB, NBA, NBH in Duroc pigs was 0.159, 0.081, 0.137; in Landrace pigs, 0.075, 0.078, 0.063; and 0.09, 0.088, 0.075 in Yorkshire pigs. Moreover, heritability of NBW, NS, OS, NBM and NM was 0.005 ~ 0.087 in Yorkshire, which was at a very low level. LAW and LBW were similar to TNB and other farrowing traits, which were found within the range of 0.078–0.128. Finally, heritability of GP in Duroc, Landrace and Yorkshire was 0.430, 0.251, and 0.303, respectively, which was considered medium heritability.

**Table 4:** Optimal model selection and estimation of heritability for three pig breeds (Duroc, Landrace, and Yorkshire).

Trait	Duroc		Landrace		Yorkshire	
	*h* ^2^	SE	*h* ^2^	SE	*h* ^2^	SE
TNB	0.159	0.027	0.075	0.010	0.094	0.006
NBA	0.081	0.033	0.078	0.011	0.088	0.006
NBH	0.137	0.026	0.063	0.010	0.075	0.006
NBW	0.082	0.022	0.056	0.008	0.087	0.005
NS	0.032	0.025	0.020	0.005	0.053	0.004
OS	0.084	0.023	0.019	0.004	0.018	0.003
NBM	0.033	0.016	0.021	0.006	0.005	0.002
NM	0.077	0.020	0.026	0.006	0.022	0.003
LBW	0.128	0.025	0.078	0.010	0.090	0.006
LAW	0.117	0.024	0.103	0.011	0.112	0.006
GP	0.430	0.031	0.251	0.016	0.303	0.009

*h*^2^, heritability; SE, standard error.

### 3.5. Effects of fixed effect factors on reproductive traits of pigs

Farm, farrowing year, breeding season, parity are fixed effect factors commonly used for estimating heritability (13, 14). These factors are relatively easy to determine by observation and collected data influence on phenotype. In the present study, these four fixed effects were selected to test 11 reproductive traits in three pig breeds from a statistical point of view using Wald Statistics (please refer to Supplementary material).

The results are shown in Table 5; Supplementary Table S7. The farm effect (since Duroc pigs originated from farms, no farm effect was observed in Duroc) impacted TNB, NBA, NBW, NS, NBM, NM, LBW, LAW, and GP in Landrace and Yorkshire (*p* < 0.05). Farrowing year did not have a significant effect on the reproductive performance of Duroc pigs, whereas it significantly affected the reproductive performance of Yorkshire pigs as well as certain traits related to reproductive performance in Landrace pigs (e.g., NBW, OS, NBM, LBW, LAW, and GP) (*p* < 0.05). The effect of breeding season on reproductive traits was evident in Yorkshire pigs, but NBM LRT results were not significant in all three pig breeds. Breeding season was not significantly affected in Duroc pigs. Moreover, parity has an important influence on reproductive performance, but it had no significant effect on reproductive traits of Duroc pigs; whereas TNB, NBA, NBH, NS, LBW, and LAW was significant affected by parity in Landrace pigs (*p* < 0.05); finally, NBM and GP were not significantly affected by parity in Yorkshire, whereas the remainder traits were significantly level (*p* < 0.05).

**Table 5:** *p* Values of the fixed effect test for the different traits in three pig breeds, i.e., DD, Duroc; LL, Landrace; YY, Yorkshire.

Fixed	Site		Year			Season			Parity		
Trait\Breed	LL	YY	DD	LL	YY	DD	LL	YY	DD	LL	YY
TNB	0.008	0.000	0.381	0.075	0.000	0.571	0.004	0.000	0.751	0.037	0.000
NBA	0.029	0.000	0.764	0.083	0.000	0.301	0.004	0.000	0.981	0.042	0.000
NBH	0.141	0.000	0.577	0.067	0.000	0.101	0.001	0.000	0.983	0.017	0.000
NBW	0.001	0.000	0.278	0.033	0.000	0.477	0.510	0.002	0.997	0.441	0.001
NS	0.000	0.000	0.154	0.230	0.000	0.113	0.007	0.000	0.625	0.019	0.000
OS	0.878	0.000	0.274	0.015	0.000	0.832	0.641	0.000	0.862	0.677	0.000
NBM	0.000	0.000	0.807	0.005	0.005	0.844	0.831	0.239	0.997	0.726	0.733
NM	0.021	0.000	0.312	0.953	0.109	0.093	0.670	0.003	0.925	0.747	0.122
LBW	0.000	0.000	0.065	0.037	0.000	0.094	0.000	0.000	0.881	0.000	0.000
LAW	0.000	0.000	0.001	0.005	0.000	0.260	0.000	0.000	0.917	0.000	0.000
GP	0.000	0.000	0.167	0.016	0.000	0.478	0.000	0.000	0.653	0.803	0.626

*p* values <0.05 were considered statistically significant.

Therefore, the majority of fixed effects had no significant impact on the reproductive traits of Duroc pigs, This result suggests that the factors influencing the reproductive performance of Duroc pigs were not attributed to the three fixed effects examined in this study. In addition, some traits of Landrace, such as NBW, OS, NBM, NM were not affected by season and party. In Yorkshire pigs, NBM is significantly influenced by site and year, but season and parity are not; other reproductive traits were affected by farm, breeding season and parity, and were considered in the multi-trait linear model.

## 4. Discussion

### 4.1. Estimation of heritability and selection of optimal model

The additive effects have long been the focus point in genetic and breeding research. Estimates of heritability for a particular trait can vary across populations due to the influence of population characteristics, models used, and datasets employed (15, 16). Moreover, it has been demonstrated that different models have biased estimates of the proportion of additive effects (17). In this study, two models were employed to estimate the heritability of 11 reproductive-related traits and assess the need to consider permanent environmental effects when calculating variance components. The heritability results obtained by the final selected model showed that the permanent environmental effects of TNB and NBH were not significant in LRT, thus model 2 was selected; however, for Landrace and Yorkshire pigs, TNB, NBA, and NBH were considered permanent environmental effects. The estimated heritability values of TNB and NBA for Landrace and Yorkshire pig breeds were found within the range of 0.075–0.09, which were lower than those of previous studies (4, 7). The heritability of TNB, NBA, and NBH was 0.125 ± 0.04 in Duroc pigs, which was considered high among the three pig breeds, and higher than the estimated level of TNB and NBA found in previous studies (16, 18) which may have included permanent environmental effects to the models. This suggests that litter size has a large interference effect among environmental factors and produces differential genetic ability in different breeds (19). Strange et al. (20) estimated heritability of weak piglet to be within the range of 0.09–0.19. The estimates of heritability obtained herein were relatively low at 0.056 ~ 0.087, thus constituting low heritability traits.

In previous studies, NS and OS have been rarely calculated separately, and the causes of NS and OS may have differed. NS may occur during sow parturition, while OS may be influenced by a combination of genetic factors, environmental conditions, and diseases during pregnancy. In this study, NS and OS were low heritability traits with the range of 0.018–0.084.This result also revealed the proportion of the additive effect of lower heritability of stillbirths (7, 21). Ye et al. showed that estimated heritability of NBM and NM in Yorkshire pigs was 0.01 (22). In contrast, the heritability of NBM and NM in Yorkshire pigs in the present study was within the range of 0.005–0.022, which was consistent with previous studies (23). However, this also suggests that there is a genetic additive effect between NM and NBM.

Birth weight is a highly heritable trait and is appropriately influenced by maternal and permanent litter effects (24). Several studies have reported that the heritability of LBW and LAW is 0.07–0.1 (16, 22), whereas other studies have shown that their heritability was higher. Shinichiro Ogawa showed that the heritability of LBW was 0.18 ± 0.01 (7), and that of LAW was 0.16 ± 0.01. Herein, LBW and LAW were within the range of 0.07–0.128 in the three pig breeds, which also confirmed the previous findings that the estimated heritability for a trait within different populations differs.

GP can serve as a significant selection indicator for breeders aiming to reduce production cycles and increase economic gains (10). In the present study, GP was found within the range of 0.251–0.430, which was similar to the results obtained by Ogawa et al. (7) and Rydhmer et al. (*h*^2^ = 0.29–0.34). Thus, the three pig breeds evaluated herein had different gestational heritability, which reveals that different pig breeds have different proportions of gestational heritability. However, it cannot be ruled out that this difference was caused by the difference in the amount of data collected.

Multiple abundant and partially novel phenotypic traits were carefully measured, and their heritability was estimated for the Duroc, Landrace, and Yorkshire pig breeds. This comprehensive investigation serves as a crucial step towards establishing a genetic parameter database specifically tailored for pig breeding in Southwest China. By expanding the scope of phenotypic traits and obtaining heritability estimates, this study provides valuable insights for future breeding programs and genetic improvement efforts in the region.

### 4.2. Genetic correlation and phenotypic correlation of reproductive traits

Genetic correlation of reproductive traits can play an important role in the selection of associated traits. The genetic correlation and phenotypic correlation between TNB and NBA were within the range of 0.839 ~ 0.969 in the three pig breeds, which indicated that TNB and NBA had high positive genetic correlation and positive phenotypic correlation; moreover, TNB, NBA, NBH, and LBW had high positive genetic correlation and phenotypic correlation. The genetic correlation map (Figures 1A–C) revealed that these traits have relatively high similarity at the genetic level (13). Roeh et al. (25) revealed a strong positive genetic correlation (0.81 ~ 0.90) between TNB and NBA in different parities of Canadian Yorkshire and Landrace pigs. Moreover, Zhang et al. (16) showed that the genetic correlation of TNB, NBA, and LBW in three pig breeds (Duroc, Landrace and Yorkshire) was within the range of 0.56–0.78, and TNB had a high positive genetic correlation with NBA and LBW (genetic correlation > 0.73). In addition, the results were similar across pig breeds, suggesting that TNB-based selection could improve both NBA and LBW.

Conversely, TNB, NBA, and NBH were negatively correlated with LAW, which is also consistent with the findings revealed in a previous study (13). Therefore, the combined selection of LBW with TNB, NBA, and NBH constitutes the opposite relationship between farrowing traits and LAW. The phenotypic correlation coefficients among NS, OS, NBM, and NM were close to 0.000, but genetic correlation coefficients were low (0.10–0.68). Interestingly, a strong positive genetic correlation was found between OS and NM, and the three pig breeds showed similar results (0.57–0.688), which has been rarely reported. These results indicated that OS and NM had a relatively high similarity in terms of affected gene. Thus, it can be speculated that the emergence of OS and NM is related to the time of first and second farrowing. If death is premature, the water in fetus tissues is absorbed leading to mummification; the yellowish epidermis produced during OS compared with NS reveals that this may be related to death in the second trimester of pregnancy. Studies have shown that age at first birth is related to farrowing intervals and dystocia, which indirectly reflects the value of associating pregnancy and farrowing traits (26). Moreover, TNB, NBA, and NBH were negatively correlated with GP in the three pig breeds evaluated herein, which was similar to the findings of a previous report (27), indicating that farrowing performance of sows can be increased by reducing GP.

### 4.3. Repeatability

Repeatability is the ratio between permanent environmental effects and phenotypic effects that can explain the proportion of phenotypic variation within a trait (28). The model of repeatability estimation was not significant as determined by LRT in Duroc pigs for NS, OS, and NBML, which may be related to the size of the dataset for Duroc pigs. Zhang et al. estimated the repeatability of TNB, NBA, LBW, GP in Duroc, Landrace, and Yorkshire breeds, and repeatability was found within the range of 0.01–0.10 (11). Ye et al. (22) estimated the repeatability of eight reproductive traits, and repeatability of each trait was within the range of 0.03–0.017. Chen et al. (11) estimated repeatability for four pig breeds to be within the range of 0.06–0.07 using animal model and restricted maximum likelihood procedures. Herein, the repeatability of the 11 reproductive traits of three pig breeds was within the range of 0.00–0.097, and repeatability of TNB, NBA, and NBH was within the range of 0.031–0.094, which were comparable to the findings of previous studies. GP-estimated repeatability of 0.044 ~ 0.069 was similar to the results reported by Cavalcante Neto et al. (29) (0.05 ~ 0.07). Differences in repeatability obtained for different pig breeds explain the degree of adaptation of different populations to different environments, which is also reflected in different reproductive traits.

### 4.4. Analysis of influencing factors of reproduction

The reproductive traits of sows are affected by both genetic and environmental factors. Among environmental factors, it can be included farm, parity, year, breeding season, and others. In contrast, genetic factors are mainly related to the genetic effects of breeds (30). In the present study, certain environmental factors affecting the reproductive traits of three pig breeds were analyzed. Among the 11 reproductive traits of the three pig breeds, farm, farrowing year, breeding season, and parity affected certain reproductive traits.

The effect of farm and farrowing year was more noticeable in each reproductive trait in Yorkshire pigs, and certain traits in Landrace pigs were significantly affected, indicating that different farming conditions have an influence on the reproductive performance of pigs (31). Although data were collected from the same company, farms have different management levels and environments, which impact reproductive traits such as TNB, NBA, NS, LBW, LAW, among others, and should be taken into account in the model when estimating heritability and genetic correlation, in order to avoid error biases of heritability and genetic correlation generated by confounding the additive variance. The farrowing year did not significantly affect the reproductive performance of Duroc pigs, but it was significant in Landrace and Yorkshire pigs, especially for NBW, OS, NBM, but not for NM, probably due to the number of NM being affected by environmental factors (32), genetic factors, and production. However, environmental factors were not the main reason for the emergence of NM in the present study.

Breeding season affected certain reproductive traits of Landrace and Yorkshire pigs. In a previous study, Untaru et al. (33) found that environmental temperature may also interact with other factors to affect sow farrowing performance. Therefore, the effect of breeding seasons on sow farrowing performance may vary among different farms (34). In addition, Almond et al. (35) described that the effect of season on the reproductive performance of sows in certain pig farms was mainly reflected in heat stress, and litter size of sows mated in the hottest months was significantly lower. Thus, the reproductive performance of sows bred during winter is usually greater than that in other seasons. Konuspayeva et al. (36) found that litter size of growing sows farrowed in autumn was the highest, and winter-farrowed litters had the lowest size. In the present study, breeding season did not affect Duroc pigs, which may be due to the small dataset. In contrast, breeding season significantly affected Landrace and Yorkshire pig in terms of TNB, NBA, and NBH, which reconfirmed the findings of Untaru et al. (33).

The impact of parity on the reproductive performance of sows remains a topic that requires further investigation (37). While factors such as farm, breeding season, and farrowing year can affect the reproductive traits of sows, breeders should pay more attention to the role of parity in influencing reproduction. Previous studies have highlighted the significance of selecting breeding sows based on their parity (38). For instance, Lucbert et al. (39) emphasized the influence of sow parity on postnatal piglet mortality, while Tummaruk et al. (40) discussed the impact of sow parity on the weaning to estrus interval in primiparous sows. In the present study, the effect of parity on the genetic parameters of different reproductive traits differed among the three pig breeds. Sow parity of Duroc pigs did not affect the value of genetic parameters, and no significance was found in the Wald test. Similarly, NBM, NM, GP for the three pig breeds were not significant, which may be due to the fact that these traits had no significant difference in each parity, and only a few related reports are available. Thus, it can be speculated that these traits are affected by maternal inheritance in absolute proportions, and parity has little effect on them. The remaining reproductive traits, including TNB, NBA, NBH, were significantly affected in Landrace and Yorkshire pigs, which was consistent with the findings of previous studies (41, 42). Moreover, sows lived longer than expected, since approximately 15% of sows reached their maximum reproductive potential at the sixth parity or later (37). Therefore, controlling parity can effectively improve production efficiency of pigs. Finally, parity significantly affected NS, and with the increase of parity, especially after the fourth parity, sow’s farrowing age increases, the uterus degenerates, litter becomes weak, and body weight increases, labor duration is prolonged, and the number of weak piglets and stillbirths gradually increases (43). Moreover, uterine atony can lead to prolonged farrowing time and increased incidence of stillbirth in pigs (44, 45).

## 5. Conclusion

In the present study, multiple models were used to test whether permanent environmental effects should be considered to the models according to LRT to adjust the bias of heritability calculation of certain reproductive traits in three pig breeds: Duroc, Landrace, and Yorkshire. TNB, NBA, NBH, NBW, NS and heritability of 11 reproduction-related traits were estimated. Differences in genetic parameters of reproductive traits were found in these populations, which indicated that the use of targeted genetic parameters in joint breeding can lead to more accurate genetic selection. With the exception of GP, which exhibited moderate heritability, the majority of reproductive traits were found to have low heritability. Moreover, the strong genetic correlation between litter-related traits is likely to play a role in multi-trait breeding, and LAW and LBW could be genetically selected by TNB or NBA. Furthermore, the factors influencing these reproductive traits were analyzed, revealing the impact of farm, farrowing year, breeding season, and parity on the evaluated sow populations. The genetic parameters, including heritability, genetic correlation, and repeatability, provide valuable insights into the genetic performance of purebred populations of the three breeds in relation to reproductive traits, thus offering data support for breeding and selection programs in pig breeding. In practical pig farming conditions, it is essential to improve management practices, and these genetic parameters are crucial for implementing optimal breeding and selection strategies.

## Data availability statement

The original contributions presented in the study are included in the article/Supplementary material, further inquiries can be directed to the corresponding authors.

## Ethics statement

This research scheme was approved by the Animal Protection and Ethics Committee of Sichuan Agricultural University (Sichuan, China, No. DKY-B20131403).

## Author contributions

YY and MG: project design and manuscript—draft writing. LZ and LS: conceptualization. JW, LN, YZ, LC, YW, HZ, and WC: methodology. XY: software. KZ, PZ, YL, and BL: data curation. All authors contributed to the article and approved the submitted version.

## Funding

This research was funded by National Key Research and Development Program of China (2021YFD1200801); Sichuan Science and Technology Program (2021YFYZ0030; 2020YFN0147; 2021ZDZX0008; and scsztd-2023-08-09); China Agriculture Research System (CARS-pig-35); and National Center of Technology Innovation for Pigs.

## Conflict of interest

YL, BL, and KZ are employed by the Sichuan Dekon Livestock Foodstuff Group. WC is employed by the National Animal Husbandry Service. HZ is employed by the Sichuan Center for Animal Disease Control.

The remaining authors declare that the research was conducted in the absence of any commercial or financial relationships that could be construed as a potential conflict of interest.

## Publisher’s note

All claims expressed in this article are solely those of the authors and do not necessarily represent those of their affiliated organizations, or those of the publisher, the editors and the reviewers. Any product that may be evaluated in this article, or claim that may be made by its manufacturer, is not guaranteed or endorsed by the publisher.
