# Supplementary material for: Estimation of genetic parameters of pig reproductive traits
Source: Front Vet Sci. 2023 Jun 21;10:1172287. doi: 10.3389/fvets.2023.1172287 (PMC10321596; doi:10.3389/fvets.2023.1172287)
Supplement: Supplementary file 1 [file Data_Sheet_1.docx]

Supplementary Material

Estimation of Genetic Parameters of Pig Reproductive Performance and Analysis of Influencing Factors

**Yitang Yang^1,2,†^, Mailin Gan^1,2,†^, Xidi Yang^1,2^, Peng Zhu^1,2^, Yi Luo^3^, Bin Liu^3^, Kangping Zhu^3^, Wenqiang Cheng^4^, Lei Chen^1,2^, Ye Zhao^1,2^, Lili Niu^1,2^, Yan Wang^1,2^, Hui Zhang^5^, Jingyong Wang^5^, Linyuan Shen^1,2*^ and Li Zhu^1,2,*^**

*** Correspondence:**Li Zhu
shenlinyuan@sicau.edu.cn (L.S.);

Linyuan Shen
shenlinyuan@sicau.edu.cn (L.S.

# Supplementary method

1.1 REML likelihood ratio test (LRT)

A general method for comparing the fit of nested models fitted by REML is the REML likelihood ratio test, or REMLRT. The REMLRT is only valid if the fixed effects are the same for both models. In ASReml-R this requires not only the same fixed effects model, but also the same parameterisation. If LR2 is the REML log-likelihood of the more general model and LR1 is the REML log-likelihood of the restricted model (that is, the REML log-likelihood under the null hypothesis), then the REMLRT is given by:

$$D=2\log\left( \frac{LR2}{LR1} \right)=2[\log\left( LR2 \right)-\log\left( LR1 \right)]$$

which is strictly positive. If r_1_ is the number of parameters estimated in model 1, then the asymptotic distribution of the REMLRT, under the restricted model is $X_{r2-r1}^{2}$.

1.2 Wald Statistics

Wald statistics are computed from an incremental sum of squares in the spirit of the approach used in classical regression analysis. For example, if we consider a very simple model with terms relating to the main effects of two qualitative factors A and B, given symbolically by

y~1+A+B

where 1 represents the constant term (µ), then the incremental sums of squares for this model can be written as the sequence

$$R(1)$$

$$R(A|1)=R(1,A)-R(1)$$

$$R(B|1,A)=R(1,A,B)-R(1,A)$$

where the R(·) operator denotes the reduction in the total sums of squares due to a model containing its argument and R(·|·) denotes the difference between the reduction in the sums of squares for any pair of (nested) models. Thus R(B|1, A) represents the difference between the reduction in sums of squares between the maximal model

$$y\sim1+A+B$$

and

$$y\sim1+A$$

Implicit in these calculations is that firstly we only compute Wald statistics for estimable functions; secondly all variance parameters are held fixed at the current REML estimates from the maximal model.

**Supplementary Tables Directory**

Supplementary Table S1. Genetic correlation and phenotype correlation in Duroc pigs.

Supplementary Table S2. Genetic correlation and phenotype correlation in Landrace pigs.

Supplementary Table S3. Genetic correlation and phenotype correlation in Yorkshire pigs.

Supplementary Table S4. Different models for estimating Duroc heritability and repeatability

Supplementary Table S5. Different models for estimating Landrace heritability and repeatability

Supplementary Table S6. Different models for estimating Yorkshire heritability and repeatability

Supplementary Table S7. Fixed effect test of different breeds.

# Supplementary Tables

## Supplementary Table S1. Genetic correlation and phenotype correlation in Duroc pigs.

|  | TNB | NBA | NBH | NBW | NS | OS | NBM | NM | LBW | LAW | GP |
| --- | --- | --- | --- | --- | --- | --- | --- | --- | --- | --- | --- |
| TNB |  | 0.859(0.037)*** | 0.829(0.047)*** | 0.528(0.122)*** | 0.384(0.154)* | 0.524(0.145)** | -0.179(0.228). | 0.359(0.143)* | 0.737(0.065)*** | -0.483(0.113)*** | -0.09(0.102). |
| NBA | 0.839(0.007)*** |  | 0.977(0.009)*** | 0.52(0.13)** | -0.055(0.176). | 0.021(0.171). | -0.132(0.239). | -0.082(0.167). | 0.888(0.031)*** | -0.486(0.117)*** | -0.112(0.106). |
| NBH | 0.781(0.01)*** | 0.946(0.003)*** |  | 0.322(0.158). | -0.032(0.179). | -0.076(0.17). | -0.175(0.234). | -0.093(0.168). | 0.945(0.02)*** | -0.35(0.132)* | -0.049(0.11). |
| NBW | 0.348(0.021)*** | 0.354(0.021)*** | 0.079(0.024)** |  | 0.1(0.203). | 0.263(0.186). | -0.185(0.268). | -0.011(0.195). | -0.018(0.175). | -0.978(0.097)*** | -0.182(0.125). |
| NS | 0.199(0.023)*** | -0.124(0.024)*** | -0.127(0.024)*** | -0.017(0.024). |  | 0.796(0.158)*** | -0.292(0.265). | 0.345(0.193). | -0.115(0.179). | -0.17(0.179). | 0.155(0.134). |
| OS | 0.179(0.024)*** | -0.128(0.024)*** | -0.143(0.024)*** | 0.038(0.024). | 0.113(0.024)*** |  | 0.154(0.277). | 0.57(0.172)** | 0.046(0.176). | 0.041(0.173). | -0.088(0.129). |
| NBM | 0.066(0.024)** | 0.079(0.024)*** | -0.064(0.024)* | -0.017(0.023). | -0.02(0.023). | -0.016(0.023). |  | -0.034(0.263). | 0.137(0.233). | 0.409(0.231). | -0.316(0.187). |
| NM | 0.336(0.022)*** | -0.077(0.024)** | -0.086(0.024)** | 0.038(0.024). | 0.055(0.024)* | 0.086(0.024)*** | 0.004(0.023). |  | -0.145(0.169). | -0.091(0.17). | -0.016(0.131). |
| LBW | 0.711(0.012)*** | 0.87(0.006)*** | 0.881(0.005)*** | 0.123(0.024)*** | -0.126(0.024)*** | -0.121(0.024)*** | 0.055(0.024). | -0.099(0.024)*** |  | -0.048(0.15). | -0.144(0.108). |
| LAW | -0.355(0.022)*** | -0.368(0.021)*** | -0.278(0.023)*** | -0.397(0.02)*** | 0.011(0.024). | 0.023(0.024). | -0.065(0.024)*** | -0.055(0.024). | 0.096(0.024)*** |  | -0.022(0.114). |
| GP | -0.168(0.027)*** | -0.182(0.027)*** | -0.147(0.027)*** | -0.102(0.026)*** | 0.054(0.026). | -0.045(0.026). | -0.074(0.025)* | -0.008(0.026). | -0.162(0.027)*** | 0.047(0.027). |  |

The upper triangle is genetic correlation, the lower triangle is phenotypic correlation。LRT ‘***’: P<0.001, ‘**’: 0.001<P<0.01, ‘*’ :0.01<P<0.05, ‘.’ :P>0.05. total number of pigs born (TNB); number of piglets born alive (NBA); number of piglets born healthy (NBH); number of piglets born weak (NBW); number of new stillborn piglets (NS); number of old stillborn piglets (OS); number of piglets born with malformation (NBM); number of mummified piglets (NM); total litter birthweight (LBW); litter average weight (LAW); duration of gestational period (GP)

## Supplementary Table S2. Genetic correlation and phenotype correlation in Landrace pigs.

|  | TNB | NBA | NBH | NBW | NS | OS | NBM | NM | LBW | LAW | GP |
| --- | --- | --- | --- | --- | --- | --- | --- | --- | --- | --- | --- |
| TNB |  | 0.969(0.004)*** | 0.939(0.008)*** | 0.631(0.037)*** | 0.384(0.154)*** | 0.237(0.091)* | 0.197(0.07)* | 0.207(0.07)** | 0.81(0.016)*** | -0.418(0.036)*** | -0.387(0.033)*** |
| NBA | 0.908(0.001)*** |  | 0.981(0.003)*** | 0.626(0.037)*** | 0.018(0.077). | -0.039(0.092). | 0.144(0.069). | -0.004(0.073). | 0.846(0.012)*** | -0.429(0.035)*** | -0.39(0.032)*** |
| NBH | 0.852(0.002)*** | 0.951(0.001)*** |  | 0.479(0.047)*** | -0.03(0.08). | -0.046(0.096). | 0.074(0.074). | -0.06(0.076). | 0.907(0.009)*** | -0.284(0.041)*** | -0.403(0.034)*** |
| NBW | 0.353(0.006)*** | 0.353(0.006)*** | 0.087(0.007)*** |  | 0.164(0.085). | -0.002(0.103). | 0.02(0.079). | 0.069(0.082). | 0.12(0.052)* | -0.814(0.027)*** | -0.107(0.043)* |
| NS | 0.199(0.023)*** | -0.089(0.007)*** | -0.107(0.007)*** | 0.03(0.007)*** |  | 0.752(0.101)*** | 0.149(0.11). | 0.459(0.105)*** | -0.043(0.077). | -0.065(0.073). | -0.026(0.064). |
| OS | 0.165(0.007)*** | -0.107(0.007)*** | -0.118(0.007)*** | 0.009(0.007). | 0.098(0.007)*** |  | 0.117(0.131). | 0.58(0.119)*** | 0.019(0.094). | 0.065(0.089). | 0.011(0.078). |
| NBM | 0.08(0.007)*** | 0.073(0.007)*** | -0.085(0.007)*** | 0.007(0.007). | 0.022(0.007)** | 0.009(0.007). |  | 0.339(0.1)** | 0.197(0.07)* | 0.004(0.067). | -0.21(0.058)*** |
| NM | 0.244(0.007)*** | -0.002(0.007). | -0.015(0.007). | 0.036(0.007)*** | 0.057(0.007)*** | 0.14(0.007)*** | 0.013(0.007)** |  | 0.007(0.074). | 0.023(0.07). | -0.09(0.061). |
| LBW | 0.765(0.003)*** | 0.86(0.002)*** | 0.872(0.002)*** | 0.133(0.007)*** | -0.104(0.007)*** | -0.119(0.007)*** | 0.028(0.007)*** | -0.016(0.007)* |  | 0.117(0.043)* | -0.394(0.033)*** |
| LAW | -0.417(0.006)*** | -0.416(0.006)*** | -0.318(0.007)*** | -0.38(0.006)*** | -0.03(0.007)*** | -0.02(0.007)** | -0.089(0.007)*** | -0.039(0.007)*** | 0.07(0.008)*** |  | 0.065(0.035). |
| GP | -0.188(0.008)*** | -0.191(0.008)*** | -0.168(0.008)*** | -0.074(0.008)*** | -0.022(0.007)** | 0.003(0.007). | -0.083(0.007)*** | -0.008(0.007). | -0.151(0.008)*** | 0.101(0.008). |  |

The upper triangle is genetic correlation, the lower triangle is phenotypic correlation。LRT ‘***’: P<0.001, ‘**’: 0.001<P<0.01, ‘*’ :0.01<P<0.05, ‘.’ :P>0.05. total number of pigs born (TNB); number of piglets born alive (NBA); number of piglets born healthy (NBH); number of piglets born weak (NBW); number of new stillborn piglets (NS); number of old stillborn piglets (OS); number of piglets born with malformation (NBM); number of mummified piglets (NM); total litter birthweight (LBW); litter average weight (LAW); duration of gestational period (GP).

## Supplementary Table S3. Genetic correlation and phenotype correlation in Yorkshire pigs.

|  | TNB | NBA | NBH | NBW | NS | OS | NBM | NM | LBW | LAW | GP |
| --- | --- | --- | --- | --- | --- | --- | --- | --- | --- | --- | --- |
| TNB |  | 0.951(0.003)*** | 0.88(0.006)*** | 0.544(0.018)*** | 0.254(0.027)*** | 0.363(0.041)*** | 0.168(0.066)*** | 0.491(0.034)*** | 0.794(0.008)*** | -0.389(0.004)*** | -0.151(0.005)*** |
| NBA | 0.888(0.001)*** |  | 0.945(0.003)*** | 0.516(0.018)*** | -0.058(0.029)*** | -0.02(0.044)*** | 0.104(0.066)*** | 0.256(0.04)*** | 0.852(0.006)*** | -0.383(0.004)*** | -0.139(0.005)*** |
| NBH | 0.79(0.002)*** | 0.915(0.001)*** |  | 0.211(0.024)*** | -0.08(0.03)*** | -0.116(0.045)*** | 0.046(0.069)*** | 0.134(0.041)*** | 0.954(0.003)*** | -0.14(0.022)*** | -0.11(0.005)*** |
| NBW | 0.403(0.003)*** | 0.394(0.004)*** | 0.004(0.004)*** |  | 0.042(0.004)*** | 0.191(0.045)*** | 0.108(0.068)* | 0.33(0.04)*** | 0.121(0.004)*** | -0.452(0.003)*** | -0.089(0.004)*** |
| NS | 0.235(0.004)*** | -0.087(0.004). | -0.112(0.004)* | 0.028(0.031). |  | 0.892(0.031)*** | 0.124(0.08)*** | 0.416(0.047)*** | -0.064(0.029)*** | -0.005(0.029)*** | -0.044(0.004)*** |
| OS | 0.174(0.004)*** | -0.116(0.004). | -0.137(0.004)* | 0.026(0.004)*** | 0.113(0.004)*** |  | 0.138(0.103)*** | 0.688(0.048)*** | -0.086(0.044)*** | -0.024(0.004)*** | -0.024(0.004)*** |
| NBM | 0.059(0.004)* | 0.05(0.004). | -0.047(0.004). | 0.01(0.004). | 0.019(0.004). | 0.017(0.004). |  | 0.286(0.097)*** | 0.101(0.067)** | 0.005(0.065)*** | -0.475(0.004)*** |
| NM | 0.239(0.004)*** | -0.038(0.004)*** | -0.063(0.004)** | 0.049(0.004)*** | 0.053(0.004)*** | 0.112(0.004)*** | 0.011(0.004)** |  | 0.148(0.04)*** | -0.042(0.004)*** | -0.007(0.004)* |
| LBW | 0.73(0.002)*** | 0.848(0.001)*** | 0.868(0.001)*** | -0.001(0.024). | -0.109(0.004)* | -0.13(0.004). | 0.012(0.004). | -0.059(0.004)*** |  | 0.13(0.004)*** | -0.097(0.005)*** |
| LAW | -0.431(0.017)*** | -0.429(0.018)*** | -0.219(0.004)*** | -0.873(0.011)*** | -0.039(0.004). | -0.098(0.043)* | -0.067(0.004). | -0.177(0.039)*** | 0.101(0.021)*** |  | 0.097(0.005)*** |
| GP | -0.207(0.017)*** | -0.198(0.017)*** | -0.176(0.018)*** | -0.115(0.019)*** | -0.048(0.024). | -0.06(0.037). | -0.057(0.004)*** | -0.104(0.034)** | -0.184(0.017)*** | 0.064(0.017)*** |  |

The upper triangle is genetic correlation, the lower triangle is phenotypic correlation。LRT ‘***’: P<0.001, ‘**’: 0.001<P<0.01, ‘*’ :0.01<P<0.05, ‘.’ :P>0.05. total number of pigs born (TNB); number of piglets born alive (NBA); number of piglets born healthy (NBH); number of piglets born weak (NBW); number of new stillborn piglets (NS); number of old stillborn piglets (OS); number of piglets born with malformation (NBM); number of mummified piglets (NM); total litter birthweight (LBW); litter average weight (LAW); duration of gestational period (GP).

## Supplementary Table S4. Different models for estimating Duroc heritability and repeatability

Mod1:$\text{rep}\text{=}\frac{\text{σ}_{\text{p}}^{\text{2}}}{\text{σ}_{\text{p}}^{\text{2}}\text{+}\text{σ}_{\text{e}}^{\text{2}}}$ Mod2: $\text{h}^{\text{2}}\text{=}\frac{\text{σ}_{\text{a}}^{\text{2}}}{\text{σ}_{\text{a}}^{\text{2}}+\text{σ}_{\text{e}}^{\text{2}}}$ mod3: $\text{h}^{\text{2}}\text{=}\frac{\text{σ}_{\text{a}}^{\text{2}}}{\text{σ}_{\text{p}}^{\text{2}}}\text{ }\text{rep=}\frac{\text{σ}_{\text{p}\text{e}}^{\text{2}}}{\text{σ}_{\text{p}}^{\text{2}}}$

where h^2^ is the heritability; rep is the repeatability;$\text{σ}_{\text{a}}^{\text{2}}$ is the additive genetic variance; $\text{σ}_{\text{p}\text{e}}^{\text{2}}$ is the permanent environmental variance; $\text{σ}_{\text{p}}^{\text{2}}$ is the total phenotypic variance, which is the sum of $\text{σ}_{\text{a}}^{\text{2}}\text{ }\text{σ}_{\text{p}\text{e}}^{\text{2}}\text{ }\text{σ}_{\text{e}}^{\text{2}}$.and $\text{σ}_{\text{e}}^{\text{2}}$ is the residual variance.

total number of pigs born (TNB); number of piglets born alive (NBA); number of piglets born healthy (NBH); number of piglets born weak (NBW); number of new stillborn piglets (NS); number of old stillborn piglets (OS); number of piglets born with malformation (NBM); number of mummified piglets (NM); total litter birthweight (LBW); litter average weight (LAW); duration of gestational period (GP)

| Phenotype | Model | Item | Component | Std.error | Target | Estimate | SE | likelihood ratio test  (mod1/mod3,  mod2/mod3) | Significance |
| --- | --- | --- | --- | --- | --- | --- | --- | --- | --- |
| TNB | mod1 | $\sigma_{p}^{2}$ | 1.188132925 | 0.211912658 | rep | 0.150530718 | 0.025075907 | 2.64949E-06  0.175510495 | ***  . |
|  |  | $\sigma_{e}^{2}$ | 6.704827 | 0.259752101 |  |  |  |  |  |
|  | mod2 | $\sigma_{a}^{2}$ | 1.274531424 | 0.240369617 | h2 | 0.158919016 | 0.027201078 |  |  |
|  |  | $\sigma_{e}^{2}$ | 6.745474356 | 0.251051623 |  |  |  |  |  |
|  | mod3 | $\sigma_{a}^{2}$ | 1.04995593 | 0.314706355 | h2 | 0.131651616 | 0.037597606 |  |  |
|  |  | $\sigma_{p}^{2}$ | 0.245239132 | 0.264482929 | rep | 0.030749984 | 0.033260475 |  |  |
|  |  | $\sigma_{e}^{2}$ | 6.680065408 | 0.257864368 |  |  |  |  |  |
| NBA | mod1 | $\sigma_{p}^{2}$ | 1.073625652 | 0.186406129 | rep | 0.156801364 | 0.025305866 | 0.000584528  0.008172163 | ***  ** |
|  |  | $\sigma_{e}^{2}$ | 5.773417135 | 0.224026817 |  |  |  |  |  |
|  | mod2 | $\sigma_{a}^{2}$ | 1.008694654 | 0.204132603 | h2 | 0.145179869 | 0.026994356 |  |  |
|  |  | $\sigma_{e}^{2}$ | 5.939201521 | 0.221148336 |  |  |  |  |  |
|  | mod3 | $\sigma_{a}^{2}$ | 0.554727069 | 0.229933172 | h2 | 0.080743179 | 0.032698705 |  |  |
|  |  | $\sigma_{p}^{2}$ | 0.542656313 | 0.23434684 | rep | 0.078986223 | 0.033976328 |  |  |
|  |  | $\sigma_{e}^{2}$ | 5.77288199 | 0.22366122 |  |  |  |  |  |
| NBH | mod1 | $\sigma_{p}^{2}$ | 0.809806285 | 0.155965529 | rep | 0.137323993 | 0.024955468 | 3.77621E-05  0.174726411 | ***  . |
|  |  | $\sigma_{e}^{2}$ | 5.087242479 | 0.197447054 |  |  |  |  |  |
|  | mod2 | $\sigma_{a}^{2}$ | 0.820196947 | 0.169189745 | h2 | 0.137423987 | 0.026206108 |  |  |
|  |  | $\sigma_{e}^{2}$ | 5.14817119 | 0.190854371 |  |  |  |  |  |
|  | mod3 | $\sigma_{a}^{2}$ | 0.657631447 | 0.219929171 | h2 | 0.110721947 | 0.035673677 |  |  |
|  |  | $\sigma_{p}^{2}$ | 0.189558597 | 0.197547294 | rep | 0.031914984 | 0.033331709 |  |  |
|  |  | $\sigma_{e}^{2}$ | 5.092295884 | 0.197076957 |  |  |  |  |  |
| NBW | mod1 | $\sigma_{p}^{2}$ | 0.052795346 | 0.013908629 | rep | 0.085438121 | 0.021923299 | 0.000581863  0.134484703 | ***  . |
|  |  | $\sigma_{e}^{2}$ | 0.565141295 | 0.021421443 |  |  |  |  |  |
|  | mod2 | $\sigma_{a}^{2}$ | 0.05086156 | 0.013987537 | h2 | 0.081916466 | 0.021699535 |  |  |
|  |  | $\sigma_{e}^{2}$ | 0.570033876 | 0.020623469 |  |  |  |  |  |
|  | mod3 | $\sigma_{a}^{2}$ | 0.03797586 | 0.016712543 | h2 | 0.061339235 | 0.026544436 |  |  |
|  |  | $\sigma_{p}^{2}$ | 0.017885934 | 0.017224064 | rep | 0.028889655 | 0.027824342 |  |  |
|  |  | $\sigma_{e}^{2}$ | 0.56325027 | 0.02130964 |  |  |  |  |  |
| NS | mod1 | $\sigma_{p}^{2}$ | 0.045119727 | 0.01253728 | rep | 0.079843028 | 0.021673043 | 0.080048268  0.042756674 | .  * |
|  |  | $\sigma_{e}^{2}$ | 0.519985681 | 0.019683634 |  |  |  |  |  |
|  | mod2 | $\sigma_{a}^{2}$ | 0.04071512 | 0.012561764 | h2 | 0.071496055 | 0.021399543 |  |  |
|  |  | $\sigma_{e}^{2}$ | 0.528758538 | 0.019151604 |  |  |  |  |  |
|  | mod3 | $\sigma_{a}^{2}$ | 0.018008181 | 0.014095383 | h2 | 0.031806223 | 0.024750598 |  |  |
|  |  | $\sigma_{p}^{2}$ | 0.028661769 | 0.01654801 | rep | 0.050622694 | 0.029148645 |  |  |
|  |  | $\sigma_{e}^{2}$ | 0.519514244 | 0.019656634 |  |  |  |  |  |
| OS | mod1 | $\sigma_{p}^{2}$ | 0.038254128 | 0.010006882 | rep | 0.087354919 | 0.022237852 | 0.055142143  0.051378993 | .  . |
|  |  | $\sigma_{e}^{2}$ | 0.399661995 | 0.015196636 |  |  |  |  |  |
|  | mod2 | $\sigma_{a}^{2}$ | 0.037225524 | 0.010530993 | h2 | 0.084139917 | 0.02290768 |  |  |
|  |  | $\sigma_{e}^{2}$ | 0.405198543 | 0.014800516 |  |  |  |  |  |
|  | mod3 | $\sigma_{a}^{2}$ | 0.018007832 | 0.012127251 | h2 | 0.040996049 | 0.027372678 |  |  |
|  |  | $\sigma_{p}^{2}$ | 0.022245411 | 0.013315423 | rep | 0.050643187 | 0.030263768 |  |  |
|  |  | $\sigma_{e}^{2}$ | 0.399004485 | 0.015157329 |  |  |  |  |  |
| NBM | mod1 | $\sigma_{p}^{2}$ | 0.005679381 | 0.002767118 | rep | 0.040591095 | 0.019640593 | 0.162297274  0.195346827 | .  . |
|  |  | $\sigma_{e}^{2}$ | 0.134237547 | 0.005024932 |  |  |  |  |  |
|  | mod2 | $\sigma_{a}^{2}$ | 0.004558597 | 0.002322409 | h2 | 0.032513211 | 0.016410574 |  |  |
|  |  | $\sigma_{e}^{2}$ | 0.135648928 | 0.004788576 |  |  |  |  |  |
|  | mod3 | $\sigma_{a}^{2}$ | 0.002668521 | 0.002830959 | h2 | 0.01905588 | 0.020160863 |  |  |
|  |  | $\sigma_{p}^{2}$ | 0.003078891 | 0.003632026 | rep | 0.021986333 | 0.025924963 |  |  |
|  |  | $\sigma_{e}^{2}$ | 0.134289187 | 0.005025195 |  |  |  |  |  |
| NM | mod1 | $\sigma_{p}^{2}$ | 0.069647468 | 0.023723686 | rep | 0.062991012 | 0.021131901 | 4.09284E-06  0.5 | ***  . |
|  |  | $\sigma_{e}^{2}$ | 1.036025644 | 0.039172124 |  |  |  |  |  |
|  | mod2 | $\sigma_{a}^{2}$ | 0.085244744 | 0.023322786 | h2 | 0.07696951 | 0.020338328 |  |  |
|  |  | $\sigma_{e}^{2}$ | 1.022268396 | 0.036627345 |  |  |  |  |  |
|  | mod3 | $\sigma_{a}^{2}$ | 0.08524467 | 0.023322827 | h2 | 0.076969444 | 0.020338362 |  |  |
|  |  | $\sigma_{p}^{2}$ | 8.70718E-08 | 0 | rep | 7.86192E-08 | 2.63608E-09 |  |  |
|  |  | $\sigma_{e}^{2}$ | 1.022268379 | 0.036627339 |  |  |  |  |  |
| LBW | mod1 | $\sigma_{p}^{2}$ | 1.538911356 | 0.308111042 | rep | 0.12779491 | 0.024293887 | 9.97296E-05  0.134521841 | ***  . |
|  |  | $\sigma_{e}^{2}$ | 10.50312816 | 0.405001121 |  |  |  |  |  |
|  | mod2 | $\sigma_{a}^{2}$ | 1.557381327 | 0.330802804 | h2 | 0.127844792 | 0.025290227 |  |  |
|  |  | $\sigma_{e}^{2}$ | 10.62443152 | 0.391303651 |  |  |  |  |  |
|  | mod3 | $\sigma_{a}^{2}$ | 1.210786527 | 0.420166567 | h2 | 0.099868374 | 0.033544209 |  |  |
|  |  | $\sigma_{p}^{2}$ | 0.422165216 | 0.387864255 | rep | 0.034821129 | 0.032043375 |  |  |
|  |  | $\sigma_{e}^{2}$ | 10.49087163 | 0.403392771 |  |  |  |  |  |
| LAW | mod1 | $\sigma_{p}^{2}$ | 0.006457431 | 0.001281151 | rep | 0.124817034 | 0.023543911 | 3.40502E-08  0.270220773 | ***  . |
|  |  | $\sigma_{e}^{2}$ | 0.04527774 | 0.001732004 |  |  |  |  |  |
|  | mod2 | $\sigma_{a}^{2}$ | 0.006072578 | 0.001304059 | h2 | 0.117496675 | 0.023669819 |  |  |
|  |  | $\sigma_{e}^{2}$ | 0.045610403 | 0.001661201 |  |  |  |  |  |
|  | mod3 | $\sigma_{a}^{2}$ | 0.005427583 | 0.00165746 | h2 | 0.105199064 | 0.030965751 |  |  |
|  |  | $\sigma_{p}^{2}$ | 0.00087911 | 0.00150922 | rep | 0.017039169 | 0.029283914 |  |  |
|  |  | $\sigma_{e}^{2}$ | 0.045286763 | 0.001726847 |  |  |  |  |  |
| GP | mod1 | $\sigma_{p}^{2}$ | 0.555478285 | 0.05393886 | rep | 0.396686788 | 0.027401213 | 0.00E+00  0.129807193 | ***  . |
|  |  | $\sigma_{e}^{2}$ | 0.84481611 | 0.033807367 |  |  |  |  |  |
|  | mod2 | $\sigma_{a}^{2}$ | 0.637355955 | 0.068984125 | h2 | 0.429631381 | 0.030579701 |  |  |
|  |  | $\sigma_{e}^{2}$ | 0.846138928 | 0.033169416 |  |  |  |  |  |
|  | mod3 | $\sigma_{a}^{2}$ | 0.549353455 | 0.098595302 | h2 | 0.377901907 | 0.055111157 |  |  |
|  |  | $\sigma_{p}^{2}$ | 0.064539687 | 0.059251091 | rep | 0.044397047 | 0.041390951 |  |  |
|  |  | $\sigma_{e}^{2}$ | 0.839800116 | 0.033335044 |  |  |  |  |  |

## Supplementary Table S5. Different models for estimating Landrace heritability and repeatability

Mod1:$\text{rep}\text{=}\frac{\text{σ}_{\text{p}}^{\text{2}}}{\text{σ}_{\text{p}}^{\text{2}}\text{+}\text{σ}_{\text{e}}^{\text{2}}}$ Mod2: $\text{h}^{\text{2}}\text{=}\frac{\text{σ}_{\text{a}}^{\text{2}}}{\text{σ}_{\text{a}}^{\text{2}}+\text{σ}_{\text{e}}^{\text{2}}}$ mod3: $\text{h}^{\text{2}}\text{=}\frac{\text{σ}_{\text{a}}^{\text{2}}}{\text{σ}_{\text{p}}^{\text{2}}}\text{ }\text{rep=}\frac{\text{σ}_{\text{p}\text{e}}^{\text{2}}}{\text{σ}_{\text{p}}^{\text{2}}}$

total number of pigs born (TNB); number of piglets born alive (NBA); number of piglets born healthy (NBH); number of piglets born weak (NBW); number of new stillborn piglets (NS); number of old stillborn piglets (OS); number of piglets born with malformation (NBM); number of mummified piglets (NM); total litter birthweight (LBW); litter average weight (LAW); duration of gestational period (GP)

| Phenotype | Model | Item | Component | Std.error | Target | Estimate | SE | likelihood ratio test  (mod1/mod3,  mod2/mod3) | Significance |
| --- | --- | --- | --- | --- | --- | --- | --- | --- | --- |
| TNB | mod1 | $\sigma_{p}^{2}$ | 1.635903845 | 0.087553127 | rep | 0.149771967 | 0.00751725 | 0.00E+00  2.59E-14 | ***  *** |
|  |  | $\sigma_{e}^{2}$ | 9.286726564 | 0.106942232 |  |  |  |  |  |
|  | mod2 | $\sigma_{a}^{2}$ | 1.675483632 | 0.107027168 | h2 | 0.149598679 | 0.008740632 |  |  |
|  |  | $\sigma_{e}^{2}$ | 9.524372136 | 0.105514421 |  |  |  |  |  |
|  | mod3 | $\sigma_{a}^{2}$ | 0.822347607 | 0.113235457 | h2 | 0.074914382 | 0.010062729 |  |  |
|  |  | $\sigma_{p}^{2}$ | 0.862117439 | 0.109714173 | rep | 0.078537342 | 0.010000615 |  |  |
|  |  | $\sigma_{e}^{2}$ | 9.292700957 | 0.106810981 |  |  |  |  |  |
| NBA | mod1 | $\sigma_{p}^{2}$ | 1.536578865 | 0.080791499 | rep | 0.15483526 | 0.007607975 | 0.00E+00  1.62E-13 | ***  *** |
|  |  | $\sigma_{e}^{2}$ | 8.387380735 | 0.09687291 |  |  |  |  |  |
|  | mod2 | $\sigma_{a}^{2}$ | 1.610348935 | 0.100116972 | h2 | 0.157741515 | 0.008910272 |  |  |
|  |  | $\sigma_{e}^{2}$ | 8.59843429 | 0.095636254 |  |  |  |  |  |
|  | mod3 | $\sigma_{a}^{2}$ | 0.782379387 | 0.108159694 | h2 | 0.078334892 | 0.01055131 |  |  |
|  |  | $\sigma_{p}^{2}$ | 0.806974222 | 0.103035302 | rep | 0.080797424 | 0.010332776 |  |  |
|  |  | $\sigma_{e}^{2}$ | 8.398269592 | 0.09684137 |  |  |  |  |  |
| NBH | mod1 | $\sigma_{p}^{2}$ | 1.217422435 | 0.07002006 | rep | 0.138279435 | 0.007522683 | 0.00E+00  1.20E-13 | ***  *** |
|  |  | $\sigma_{e}^{2}$ | 7.58665199 | 0.087545204 |  |  |  |  |  |
|  | mod2 | $\sigma_{a}^{2}$ | 1.21350849 | 0.083683809 | h2 | 0.134735022 | 0.008603577 |  |  |
|  |  | $\sigma_{e}^{2}$ | 7.793121491 | 0.086170197 |  |  |  |  |  |
|  | mod3 | $\sigma_{a}^{2}$ | 0.558981492 | 0.086140058 | h2 | 0.063199932 | 0.00954979 |  |  |
|  |  | $\sigma_{p}^{2}$ | 0.688432591 | 0.087776581 | rep | 0.077836017 | 0.009913809 |  |  |
|  |  | $\sigma_{e}^{2}$ | 7.59723934 | 0.087551812 |  |  |  |  |  |
| NBW | mod1 | $\sigma_{p}^{2}$ | 0.066746372 | 0.005027976 | rep | 0.090741116 | 0.006649313 | 0.00E+00  3.40E-06 | ***  *** |
|  |  | $\sigma_{e}^{2}$ | 0.668822852 | 0.007520063 |  |  |  |  |  |
|  | mod2 | $\sigma_{a}^{2}$ | 0.063116268 | 0.005398065 | h2 | 0.085066295 | 0.006979748 |  |  |
|  |  | $\sigma_{e}^{2}$ | 0.678849371 | 0.007246279 |  |  |  |  |  |
|  | mod3 | $\sigma_{a}^{2}$ | 0.041347986 | 0.006025303 | h2 | 0.056085868 | 0.008030742 |  |  |
|  |  | $\sigma_{p}^{2}$ | 0.027344389 | 0.006177933 | rep | 0.037090894 | 0.008389577 |  |  |
|  |  | $\sigma_{e}^{2}$ | 0.668534098 | 0.007501314 |  |  |  |  |  |
| NS | mod1 | $\sigma_{p}^{2}$ | 0.031432234 | 0.0045812 | rep | 0.04461178 | 0.006454265 | 6.11E-09  0.000311571 | ***  *** |
|  |  | $\sigma_{e}^{2}$ | 0.673140272 | 0.007613023 |  |  |  |  |  |
|  | mod2 | $\sigma_{a}^{2}$ | 0.021432845 | 0.003545114 | h2 | 0.030368532 | 0.004974483 |  |  |
|  |  | $\sigma_{e}^{2}$ | 0.684325506 | 0.007059191 |  |  |  |  |  |
|  | mod3 | $\sigma_{a}^{2}$ | 0.014451911 | 0.003589811 | h2 | 0.020496333 | 0.005068151 |  |  |
|  |  | $\sigma_{p}^{2}$ | 0.017644188 | 0.005216145 | rep | 0.025023761 | 0.007390399 |  |  |
|  |  | $\sigma_{e}^{2}$ | 0.673001259 | 0.007605901 |  |  |  |  |  |
| OS | mod1 | $\sigma_{p}^{2}$ | 0.012097481 | 0.003194607 | rep | 0.023849637 | 0.006283908 | 4.42315E-05  0.104840978 | ***  . |
|  |  | $\sigma_{e}^{2}$ | 0.49514217 | 0.005605338 |  |  |  |  |  |
|  | mod2 | $\sigma_{a}^{2}$ | 0.009642498 | 0.002233167 | h2 | 0.018987335 | 0.004376013 |  |  |
|  |  | $\sigma_{e}^{2}$ | 0.498195889 | 0.005090442 |  |  |  |  |  |
|  | mod3 | $\sigma_{a}^{2}$ | 0.007871762 | 0.00243359 | h2 | 0.015505116 | 0.004779099 |  |  |
|  |  | $\sigma_{p}^{2}$ | 0.004807803 | 0.00366013 | rep | 0.009469993 | 0.007209312 |  |  |
|  |  | $\sigma_{e}^{2}$ | 0.495008493 | 0.005601668 |  |  |  |  |  |
| NBM | mod1 | $\sigma_{p}^{2}$ | 0.013120922 | 0.001567496 | rep | 0.056639823 | 0.00668987 | 1.92619E-07  3.3432E-06 | ***  *** |
|  |  | $\sigma_{e}^{2}$ | 0.218534505 | 0.002486147 |  |  |  |  |  |
|  | mod2 | $\sigma_{a}^{2}$ | 0.009302222 | 0.001349691 | h2 | 0.040013056 | 0.005723761 |  |  |
|  |  | $\sigma_{e}^{2}$ | 0.223177457 | 0.002339021 |  |  |  |  |  |
|  | mod3 | $\sigma_{a}^{2}$ | 0.004953075 | 0.001303219 | h2 | 0.021357169 | 0.005593234 |  |  |
|  |  | $\sigma_{p}^{2}$ | 0.008408532 | 0.001822248 | rep | 0.036256757 | 0.007841689 |  |  |
|  |  | $\sigma_{e}^{2}$ | 0.218554676 | 0.002485382 |  |  |  |  |  |
| NM | mod1 | $\sigma_{p}^{2}$ | 0.017377241 | 0.002772612 | rep | 0.038475714 | 0.006103749 | 2.71103E-09  0.033021487 | ***  * |
|  |  | $\sigma_{e}^{2}$ | 0.434264563 | 0.004848447 |  |  |  |  |  |
|  | mod2 | $\sigma_{a}^{2}$ | 0.015254367 | 0.002369428 | h2 | 0.033680774 | 0.005172942 |  |  |
|  |  | $\sigma_{e}^{2}$ | 0.437655856 | 0.004531376 |  |  |  |  |  |
|  | mod3 | $\sigma_{a}^{2}$ | 0.011634985 | 0.002661 | h2 | 0.02571997 | 0.005846705 |  |  |
|  |  | $\sigma_{p}^{2}$ | 0.006288569 | 0.003347915 | rep | 0.013901335 | 0.00740282 |  |  |
|  |  | $\sigma_{e}^{2}$ | 0.434448079 | 0.004847898 |  |  |  |  |  |
| LBW | mod1 | $\sigma_{p}^{2}$ | 2.805502831 | 0.150141024 | rep | 0.148315885 | 0.007449866 | 0.00E+00  2.07612E-13 | ***  *** |
|  |  | $\sigma_{e}^{2}$ | 16.1102244 | 0.185050382 |  |  |  |  |  |
|  | mod2 | $\sigma_{a}^{2}$ | 2.823141733 | 0.181206124 | h2 | 0.145894748 | 0.008589411 |  |  |
|  |  | $\sigma_{e}^{2}$ | 16.52739537 | 0.182389142 |  |  |  |  |  |
|  | mod3 | $\sigma_{a}^{2}$ | 1.477374479 | 0.19665209 | h2 | 0.077737899 | 0.010080374 |  |  |
|  |  | $\sigma_{p}^{2}$ | 1.393934847 | 0.188050231 | rep | 0.073347393 | 0.009909052 |  |  |
|  |  | $\sigma_{e}^{2}$ | 16.13324964 | 0.185007545 |  |  |  |  |  |
| LAW | mod1 | $\sigma_{p}^{2}$ | 0.008020569 | 0.000397782 | rep | 0.160282982 | 0.007389123 | 0.00E+00  4.31121E-10 | ***  *** |
|  |  | $\sigma_{e}^{2}$ | 0.042019482 | 0.00048068 |  |  |  |  |  |
|  | mod2 | $\sigma_{a}^{2}$ | 0.008397516 | 0.00048924 | h2 | 0.163848101 | 0.008618427 |  |  |
|  |  | $\sigma_{e}^{2}$ | 0.042854322 | 0.000472432 |  |  |  |  |  |
|  | mod3 | $\sigma_{a}^{2}$ | 0.005208649 | 0.000580772 | h2 | 0.103392733 | 0.011095216 |  |  |
|  |  | $\sigma_{p}^{2}$ | 0.003115453 | 0.000504609 | rep | 0.061842368 | 0.010070328 |  |  |
|  |  | $\sigma_{e}^{2}$ | 0.042053216 | 0.000479896 |  |  |  |  |  |
| GP | mod1 | $\sigma_{p}^{2}$ | 0.565741942 | 0.019840944 | rep | 0.27995936 | 0.008173203 | 0.00E+00  0.000144122 | ***  *** |
|  |  | $\sigma_{e}^{2}$ | 1.455058297 | 0.017111878 |  |  |  |  |  |
|  | mod2 | $\sigma_{a}^{2}$ | 0.627641334 | 0.025787704 | h2 | 0.298649695 | 0.009669306 |  |  |
|  |  | $\sigma_{e}^{2}$ | 1.473955768 | 0.016837781 |  |  |  |  |  |
|  | mod3 | $\sigma_{a}^{2}$ | 0.517585966 | 0.03668518 | h2 | 0.250596565 | 0.015713741 |  |  |
|  |  | $\sigma_{p}^{2}$ | 0.088662874 | 0.024175932 | rep | 0.042927384 | 0.011860523 |  |  |
|  |  | $\sigma_{e}^{2}$ | 1.459166407 | 0.017068112 |  |  |  |  |  |

## Supplementary Table S6. Different models for estimating Yorkshire heritability and repeatability

Mod1:$\text{rep}\text{=}\frac{\text{σ}_{\text{p}}^{\text{2}}}{\text{σ}_{\text{p}}^{\text{2}}\text{+}\text{σ}_{\text{e}}^{\text{2}}}$ Mod2: $\text{h}^{\text{2}}\text{=}\frac{\text{σ}_{\text{a}}^{\text{2}}}{\text{σ}_{\text{a}}^{\text{2}}+\text{σ}_{\text{e}}^{\text{2}}}$ mod3: $\text{h}^{\text{2}}\text{=}\frac{\text{σ}_{\text{a}}^{\text{2}}}{\text{σ}_{\text{p}}^{\text{2}}}\text{ }\text{rep=}\frac{\text{σ}_{\text{p}\text{e}}^{\text{2}}}{\text{σ}_{\text{p}}^{\text{2}}}$

total number of pigs born (TNB); number of piglets born alive (NBA); number of piglets born healthy (NBH); number of piglets born weak (NBW); number of new stillborn piglets (NS); number of old stillborn piglets (OS); number of piglets born with malformation (NBM); number of mummified piglets (NM); total litter birthweight (LBW); litter average weight (LAW); duration of gestational period (GP)

| Phenotype | Model | | Item | Component | Std.error | Target | Estimate | SE | likelihood ratio test  (mod1/mod3,mod2/mod) | Significance |
| --- | --- | --- | --- | --- | --- | --- | --- | --- | --- | --- |
| TNB | | mod1 | $\sigma_{p}^{2}$ | 1.908800764 | 0.046911968 | rep | 0.184299715 | 0.004126125 | 0.00E+00  0.00E+00 | ***  *** |
|  |  |  | $\sigma_{e}^{2}$ | 8.448245978 | 0.052384583 |  |  |  |  |  |
|  |  | mod2 | $\sigma_{a}^{2}$ | 2.110746632 | 0.063545362 | h2 | 0.195640721 | 0.005151303 |  |  |
|  |  |  | $\sigma_{e}^{2}$ | 8.678145508 | 0.052224639 |  |  |  |  |  |
|  |  | mod3 | $\sigma_{a}^{2}$ | 0.981704544 | 0.066906215 | h2 | 0.094257404 | 0.006190046 |  |  |
|  |  |  | $\sigma_{p}^{2}$ | 0.977142312 | 0.05805785 | rep | 0.093819366 | 0.005607149 |  |  |
|  |  |  | $\sigma_{e}^{2}$ | 8.456298309 | 0.052307401 |  |  |  |  |  |
| NBA | | mod1 | $\sigma_{p}^{2}$ | 1.689281419 | 0.042208186 | rep | 0.181891814 | 0.004149812 | 0.00E+00  0.00E+00 | ***  *** |
|  |  |  | $\sigma_{e}^{2}$ | 7.598005267 | 0.047205954 |  |  |  |  |  |
|  |  | mod2 | $\sigma_{a}^{2}$ | 1.869101523 | 0.057225339 | h2 | 0.193064593 | 0.00518357 |  |  |
|  |  |  | $\sigma_{e}^{2}$ | 7.812122248 | 0.047091288 |  |  |  |  |  |
|  |  | mod3 | $\sigma_{a}^{2}$ | 0.820805383 | 0.058726482 | h2 | 0.08791136 | 0.006080684 |  |  |
|  |  |  | $\sigma_{p}^{2}$ | 0.909093748 | 0.052236685 | rep | 0.097367377 | 0.005618955 |  |  |
|  |  |  | $\sigma_{e}^{2}$ | 7.60683944 | 0.047158019 |  |  |  |  |  |
| NBH | | mod1 | $\sigma_{p}^{2}$ | 1.306964999 | 0.03489049 | rep | 0.167048876 | 0.004122614 | 0.00E+00  0.00E+00 | ***  *** |
|  |  |  | $\sigma_{e}^{2}$ | 6.516882916 | 0.040479792 |  |  |  |  |  |
|  |  | mod2 | $\sigma_{a}^{2}$ | 1.405207964 | 0.046279895 | h2 | 0.173147428 | 0.005093373 |  |  |
|  |  |  | $\sigma_{e}^{2}$ | 6.710465385 | 0.040298971 |  |  |  |  |  |
|  |  | mod3 | $\sigma_{a}^{2}$ | 0.589447294 | 0.045825933 | h2 | 0.075009225 | 0.005672608 |  |  |
|  |  |  | $\sigma_{p}^{2}$ | 0.741951925 | 0.042903282 | rep | 0.094415972 | 0.005467674 |  |  |
|  |  | | $\sigma_{e}^{2}$ | 6.526931449 | 0.04046639 |  |  |  |  |  |
| NBW | mod1 | | $\sigma_{p}^{2}$ | 0.196165422 | 0.005813354 | rep | 0.133710989 | 0.003748337 | 0.00E+00  0.00E+00 | ***  *** |
|  |  | | $\sigma_{e}^{2}$ | 1.270919848 | 0.007716244 |  |  |  |  |  |
|  | mod2 | | $\sigma_{a}^{2}$ | 0.199797853 | 0.007180033 | h2 | 0.134052045 | 0.004441679 |  |  |
|  |  | | $\sigma_{e}^{2}$ | 1.29065202 | 0.007530708 |  |  |  |  |  |
|  | mod3 | | $\sigma_{a}^{2}$ | 0.127355724 | 0.008281286 | h2 | 0.086744025 | 0.005449727 |  |  |
|  |  | | $\sigma_{p}^{2}$ | 0.070532109 | 0.00707331 | rep | 0.04804055 | 0.004844543 |  |  |
|  |  | | $\sigma_{e}^{2}$ | 1.270290809 | 0.007682151 |  |  |  |  |  |
| NS | mod1 | | $\sigma_{p}^{2}$ | 0.064234534 | 0.00303457 | rep | 0.077485967 | 0.003584383 | 0.00E+00  1.34215E-10 | ***  *** |
|  |  | | $\sigma_{e}^{2}$ | 0.76474826 | 0.004645486 |  |  |  |  |  |
|  | mod2 | | $\sigma_{a}^{2}$ | 0.060743597 | 0.003223663 | h2 | 0.072719492 | 0.003724448 |  |  |
|  |  | | $\sigma_{e}^{2}$ | 0.774570236 | 0.004421576 |  |  |  |  |  |
|  | mod3 | | $\sigma_{a}^{2}$ | 0.044240158 | 0.0036316 | h2 | 0.05321164 | 0.004287051 |  |  |
|  |  | | $\sigma_{p}^{2}$ | 0.022601488 | 0.003615289 | rep | 0.027184854 | 0.004356335 |  |  |
|  |  | | $\sigma_{e}^{2}$ | 0.76455837 | 0.004632108 |  |  |  |  |  |
| OS | mod1 | | $\sigma_{p}^{2}$ | 0.017975921 | 0.001756112 | rep | 0.03349635 | 0.003257527 | 0.00E+00  5.01731E-06 | ***  *** |
|  |  | | $\sigma_{e}^{2}$ | 0.518677212 | 0.003118341 |  |  |  |  |  |
|  | mod2 | | $\sigma_{a}^{2}$ | 0.013012308 | 0.001348666 | h2 | 0.024214827 | 0.002489674 |  |  |
|  |  | | $\sigma_{e}^{2}$ | 0.524357149 | 0.002880498 |  |  |  |  |  |
|  | mod3 | | $\sigma_{a}^{2}$ | 0.009618224 | 0.001358429 | h2 | 0.017915072 | 0.002518639 |  |  |
|  |  | | $\sigma_{p}^{2}$ | 0.008575376 | 0.001968661 | rep | 0.015972644 | 0.003666367 |  |  |
|  |  | | $\sigma_{e}^{2}$ | 0.518685298 | 0.003115456 |  |  |  |  |  |
| NBM | mod1 | | $\sigma_{p}^{2}$ | 0.001685839 | 0.000254047 | rep | 0.02136359 | 0.003213227 | 2.61897E-05  5.94765E-07 | ***  *** |
|  |  | | $\sigma_{e}^{2}$ | 0.077225962 | 0.000465043 |  |  |  |  |  |
|  | mod2 | | $\sigma_{a}^{2}$ | 0.000710147 | 0.000145874 | h2 | 0.008994737 | 0.001844026 |  |  |
|  |  | | $\sigma_{e}^{2}$ | 0.078241282 | 0.00042186 |  |  |  |  |  |
|  | mod3 | | $\sigma_{a}^{2}$ | 0.000382952 | 0.000124663 | h2 | 0.004852134 | 0.001578368 |  |  |
|  |  | | $\sigma_{p}^{2}$ | 0.001333805 | 0.000272274 | rep | 0.016899767 | 0.003446812 |  |  |
|  |  | | $\sigma_{e}^{2}$ | 0.077207723 | 0.000464904 |  |  |  |  |  |
| NM | mod1 | | $\sigma_{p}^{2}$ | 0.023867896 | 0.001960729 | rep | 0.040508001 | 0.00330621 | 0.00E+00  1.9647E-07 | ***  *** |
|  |  | | $\sigma_{e}^{2}$ | 0.56534646 | 0.003402688 |  |  |  |  |  |
|  | mod2 | | $\sigma_{a}^{2}$ | 0.018040445 | 0.001631564 | h2 | 0.030557145 | 0.002733278 |  |  |
|  |  | | $\sigma_{e}^{2}$ | 0.572343404 | 0.003168929 |  |  |  |  |  |
|  | mod3 | | $\sigma_{a}^{2}$ | 0.012765951 | 0.001653885 | h2 | 0.021655536 | 0.002789161 |  |  |
|  |  | | $\sigma_{p}^{2}$ | 0.011193722 | 0.002228887 | rep | 0.018988483 | 0.003780787 |  |  |
|  |  | | $\sigma_{e}^{2}$ | 0.565540931 | 0.003399666 |  |  |  |  |  |
| LBW | mod1 | | $\sigma_{p}^{2}$ | 2.779399549 | 0.071083066 | rep | 0.173173569 | 0.004071323 | 0.00E+00  0.00E+00 | ***  *** |
|  |  | | $\sigma_{e}^{2}$ | 13.27039121 | 0.082046827 |  |  |  |  |  |
|  | mod2 | | $\sigma_{a}^{2}$ | 3.018138149 | 0.094573761 | h2 | 0.18127941 | 0.005033658 |  |  |
|  |  | | $\sigma_{e}^{2}$ | 13.63095699 | 0.08157161 |  |  |  |  |  |
|  | mod3 | | $\sigma_{a}^{2}$ | 1.445590831 | 0.100339082 | h2 | 0.089557169 | 0.006003307 |  |  |
|  |  | | $\sigma_{p}^{2}$ | 1.412145144 | 0.088044356 | rep | 0.087485144 | 0.00548371 |  |  |
|  |  | | $\sigma_{e}^{2}$ | 13.28380632 | 0.081942013 |  |  |  |  |  |
| LAW | mod1 | | $\sigma_{p}^{2}$ | 0.006766037 | 0.000164682 | rep | 0.180639364 | 0.004013122 | 0.00E+00  0.00E+00 | ***  *** |
|  |  | | $\sigma_{e}^{2}$ | 0.030690011 | 0.000188792 |  |  |  |  |  |
|  | mod2 | | $\sigma_{a}^{2}$ | 0.007161171 | 0.000215235 | h2 | 0.185809559 | 0.004924418 |  |  |
|  |  | | $\sigma_{e}^{2}$ | 0.031379207 | 0.000186733 |  |  |  |  |  |
|  | mod3 | | $\sigma_{a}^{2}$ | 0.004209193 | 0.000248912 | h2 | 0.112042885 | 0.006321768 |  |  |
|  |  | | $\sigma_{p}^{2}$ | 0.002617407 | 0.000201078 | rep | 0.069671753 | 0.00540091 |  |  |
|  |  | | $\sigma_{e}^{2}$ | 0.030741092 | 0.000188436 |  |  |  |  |  |
| GP | mod1 | | $\sigma_{p}^{2}$ | 0.751014091 | 0.012041359 | rep | 0.359908203 | 0.004342982 |  |  |
|  |  | | $\sigma_{e}^{2}$ | 1.335668248 | 0.008474853 |  |  |  | 0.00E+00  0.00E+00 | ***  *** |
|  | mod2 | | $\sigma_{a}^{2}$ | 0.865036929 | 0.016746158 | h2 | 0.38831729 | 0.005253641 |  |  |
|  |  | | $\sigma_{e}^{2}$ | 1.362618012 | 0.008481315 |  |  |  |  |  |
|  | mod3 | | $\sigma_{a}^{2}$ | 0.648541486 | 0.023793974 | h2 | 0.302904324 | 0.009417315 |  |  |
|  |  | | $\sigma_{p}^{2}$ | 0.147737969 | 0.01424561 | rep | 0.069001707 | 0.006844237 |  |  |
|  |  | | $\sigma_{e}^{2}$ | 1.344797563 | 0.008493548 |  |  |  |  |  |

## Supplementary Table S7. Fixed effect test of different breeds.

| Trait | Breed | DD | | |  | LL | | | |  | YY | | | |
| --- | --- | --- | --- | --- | --- | --- | --- | --- | --- | --- | --- | --- | --- | --- |
|  | Item | Year | Season | Parity |  | Site | Year | Season | Parity |  | Site | Year | Season | Parity |
|  | Df1 | 5 | 3 | 8 |  | 10 | 6 | 3 | 9 |  | 10 | 6 | 3 | 10 |
| TNB | Sum of Sq2 | 26.459 | 6.025 | 40.468 |  | 238.420 | 68.774 | 40.034 | 160.554 |  | 1116.464 | 325.582 | 60.519 | 999.999 |
|  | Wald statistic3 | 5.292 | 2.008 | 5.058 |  | 23.842 | 11.462 | 13.345 | 17.839 |  | 111.646 | 54.264 | 20.173 | 100.000 |
|  | Pr(Chisq)4 | 0.381 | 0.571 | 0.751 |  | 0.008 | 0.075 | 0.004 | 0.037 |  | 0.000 | 0.000 | 0.000 | 0.000 |
| NBA | Sum of Sq | 12.918 | 10.978 | 15.969 |  | 199.904 | 67.149 | 40.481 | 156.976 |  | 850.231 | 148.753 | 160.909 | 1053.493 |
|  | Wald statistic | 2.584 | 3.659 | 1.996 |  | 19.990 | 11.191 | 13.494 | 17.442 |  | 85.023 | 24.792 | 53.636 | 105.349 |
|  | Pr(Chisq) | 0.764 | 0.301 | 0.981 |  | 0.029 | 0.083 | 0.004 | 0.042 |  | 0.000 | 0.000 | 0.000 | 0.000 |
| NBH | Sum of Sq | 19.050 | 18.718 | 15.327 |  | 147.605 | 70.794 | 51.909 | 181.700 |  | 640.581 | 156.054 | 208.870 | 1082.794 |
|  | Wald statistic | 3.810 | 6.239 | 1.916 |  | 14.761 | 11.799 | 17.303 | 20.189 |  | 64.058 | 26.009 | 69.623 | 108.279 |
|  | Pr(Chisq) | 0.577 | 0.101 | 0.983 |  | 0.141 | 0.067 | 0.001 | 0.017 |  | 0.000 | 0.000 | 0.000 | 0.000 |
| NBW | Sum of Sq | 31.511 | 7.477 | 9.611 |  | 302.987 | 82.265 | 6.932 | 80.589 |  | 753.209 | 273.294 | 45.074 | 287.546 |
|  | Wald statistic | 6.302 | 2.492 | 1.201 |  | 30.299 | 13.711 | 2.311 | 8.954 |  | 75.321 | 45.549 | 15.025 | 28.755 |
|  | Pr(Chisq) | 0.278 | 0.477 | 0.997 |  | 0.001 | 0.033 | 0.510 | 0.441 |  | 0.000 | 0.000 | 0.002 | 0.001 |
| NS | Sum of Sq | 40.160 | 17.918 | 49.622 |  | 409.392 | 48.635 | 36.799 | 178.214 |  | 1468.129 | 303.074 | 104.114 | 958.142 |
|  | Wald statistic | 8.032 | 5.973 | 6.203 |  | 40.939 | 8.106 | 12.266 | 19.802 |  | 146.813 | 50.512 | 34.705 | 95.814 |
|  | Pr(Chisq) | 0.154 | 0.113 | 0.625 |  | 0.000 | 0.230 | 0.007 | 0.019 |  | 0.000 | 0.000 | 0.000 | 0.000 |
| OS | Sum of Sq | 31.725 | 2.622 | 31.541 |  | 51.860 | 95.095 | 5.050 | 59.508 |  | 368.303 | 326.139 | 82.745 | 442.724 |
|  | Wald statistic | 6.345 | 0.874 | 3.943 |  | 5.186 | 15.849 | 1.683 | 6.612 |  | 36.830 | 54.356 | 27.582 | 44.272 |
|  | Pr(Chisq) | 0.274 | 0.832 | 0.862 |  | 0.878 | 0.015 | 0.641 | 0.677 |  | 0.000 | 0.000 | 0.000 | 0.000 |
| NBM | Sum of Sq | 11.465 | 2.465 | 9.312 |  | 323.695 | 110.153 | 2.626 | 55.279 |  | 377.997 | 112.171 | 12.636 | 69.191 |
|  | Wald statistic | 2.293 | 0.822 | 1.164 |  | 32.370 | 18.359 | 0.875 | 6.142 |  | 37.800 | 18.695 | 4.212 | 6.919 |
|  | Pr(Chisq) | 0.807 | 0.844 | 0.997 |  | 0.000 | 0.005 | 0.831 | 0.726 |  | 0.000 | 0.005 | 0.239 | 0.733 |
| NM | Sum of Sq | 29.700 | 19.216 | 25.169 |  | 209.622 | 9.596 | 4.661 | 53.328 |  | 549.985 | 62.383 | 42.021 | 152.851 |
|  | Wald statistic | 5.940 | 6.405 | 3.146 |  | 20.962 | 1.599 | 1.554 | 5.925 |  | 54.999 | 10.397 | 14.007 | 15.285 |
|  | Pr(Chisq) | 0.312 | 0.093 | 0.925 |  | 0.021 | 0.953 | 0.670 | 0.747 |  | 0.000 | 0.109 | 0.003 | 0.122 |
| LBW | Sum of Sq | 51.866 | 19.182 | 29.763 |  | 417.583 | 80.456 | 54.276 | 598.093 |  | 1715.814 | 222.521 | 188.455 | 2171.938 |
|  | Wald statistic | 10.373 | 6.394 | 3.720 |  | 41.758 | 13.409 | 18.092 | 66.455 |  | 171.581 | 37.087 | 62.818 | 217.194 |
|  | Pr(Chisq) | 0.065 | 0.094 | 0.881 |  | 0.000 | 0.037 | 0.000 | 0.000 |  | 0.000 | 0.000 | 0.000 | 0.000 |
| LAW | Sum of Sq | 99.057 | 12.030 | 26.083 |  | 867.058 | 111.579 | 57.358 | 461.491 |  | 2686.661 | 440.938 | 298.756 | 1320.391 |
|  | Wald statistic | 19.811 | 4.010 | 3.260 |  | 86.706 | 18.596 | 19.119 | 51.277 |  | 268.666 | 73.490 | 99.585 | 132.039 |
|  | Pr(Chisq) | 0.001 | 0.260 | 0.917 |  | 0.000 | 0.005 | 0.000 | 0.000 |  | 0.000 | 0.000 | 0.000 | 0.000 |
| GP | Sum of Sq | 39.094 | 7.449 | 47.613 |  | 363.237 | 94.067 | 77.075 | 48.125 |  | 1775.528 | 742.394 | 412.200 | 80.319 |
|  | Wald statistic | 7.819 | 2.483 | 5.952 |  | 36.324 | 15.678 | 25.692 | 5.347 |  | 177.553 | 123.732 | 137.400 | 8.032 |
|  | Pr(Chisq) | 0.167 | 0.478 | 0.653 |  | 0.000 | 0.016 | 0.000 | 0.803 |  | 0.000 | 0.000 | 0.000 | 0.626 |

1. Df represents the degrees of freedom of the corresponding model items.

2. Sum of Sq represents the sum of squares of the terms,.

3. Wald statistic represents the sum of squares and residuals of the terms (MS) ratio of mean squares, similar to the F value of ANOVA.

4. Pr(Chisq) gives an intuitive significance level for each item.

5. total number of pigs born (TNB); number of piglets born alive (NBA); number of piglets born healthy (NBH); number of piglets born weak (NBW); number of new stillborn piglets (NS); number of old stillborn piglets (OS); number of piglets born with malformation (NBM); number of mummified piglets (NM); total litter birthweight (LBW); litter average weight (LAW); duration of gestational period (GP).
